# Effect of sub-micron deformations at opposing strain rates on the micromagnetic behaviour of non-oriented electrical steel

**DOI:** 10.1038/s41467-024-53346-7

**Published:** 2024-10-18

**Authors:** Kieran Winter, Zhirong Liao, Erik Abbá, Jose A. Robles Linares, Dragos Axinte

**Affiliations:** Rolls-Royce University Technology Centre in Manufacturing and On-Wing Technology, Faculty of Engineering, University of Nottingham, Nottingham, UK

**Keywords:** Magnetic properties and materials, Magnetic properties and materials

## Abstract

We are entering an era of re-electrification, seeking high-power density electrical machines with minimal resource use. Significant performance gains in electrical machines have been achieved through precise manufacturing processes, including the shaping/cutting of soft magnetic materials. However, most studies have evaluated magnetic performance at a macro level, focusing on components, while the fundamental mechanisms, e.g., how the micromagnetic behaviour is affected by mechanical interference, remain unclear. In this study, we examine the impact of sub-micron deformations at opposing strain rates (10^−2^ to 10^1^ s^−1^) on the micromagnetic behaviour of soft magnetic non-oriented electrical steel. Using a diamond probe to indent within a single grain of polycrystalline material at different velocities, we induce quasi-static and dynamic mechanical loading. Our analysis, employing magnetic force microscopy, transmission Kikuchi diffraction, and scanning transmission electron microscopy with a pixelated detector, reveals that magnetic texture disturbances rely on the time-dependent dislocation dynamics of the Fe-BCC material. Additionally, we compress micro-pillars to further investigate these effects under bulk-isolated deformation. These findings highlight the importance of considering even ultra-small loads, such as nano-indentations and micro-pillar compressions, in the manufacturing of next-generation electric machines, as they can affect magnetic texture and performance.

## Abstract (web summary)

Mechanical deformations affect magnetic properties of electrical steels, impacting machine efficiency. Here, authors demonstrate that sub-micron deformations at different strain rates alter micromagnetic behaviour of non-oriented electrical steel due to time-dependent dislocation dynamics.

## Introduction

With the global trend towards the use of cleaner energy sources and, the goal to limit the utilisation of fossil fuels^1^, the expansion of electric propulsion is becoming progressively more viable^2^. This trend is occurring in sectors spanning society, including: ‘last-mile logistics’ such as E-scooters^3^ for personal commuting; the automotive sector – driven in large part through legislative action; and the aerospace sector, where future economic security of companies depends on their ability to embrace sustainability^4^.

With limited resources, the importance of energy efficiency in all the aforementioned sectors is paramount for meeting future demands and, one way to tackle this is by producing ever-more efficient (e.g., minimal magnetic losses) electrical propulsion systems.

**Fig. 1:**
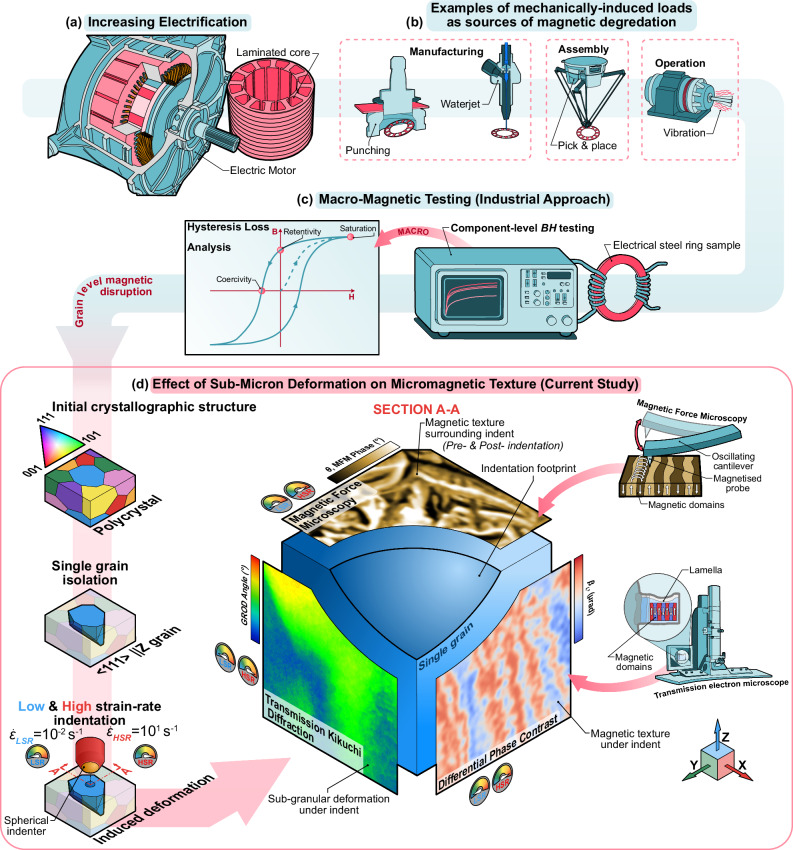
Scope of the investigation. **a** Electric motor application. **b** Example sources of mechanically induced loads experienced by electrical steel laminations from manufacture to assembly, and its operational life. **c** Conventional macro-level BH magnetic testing completed at the component level which is commonly used for measuring the magnetic performance of electrical steel laminations. **d** Schematic overview of the experimental procedure. Showing the selection of a single grain through EBSD inverse pole figure mapping (with associated stereographic key) and the induced deformation at low (LSR) and high (HSR) strain rates. A schematic representation of the data cube is shown with magnetic force microscopy (MFM) on the free-surface (||XY) and transmission Kikuchi diffraction (TKD) and differential phase contrast being used to investigate the material deformation and magnetic domain structure, respectively.

Focusing on the design and manufacture of motors, electrical steel laminations containing Fe with a low addition of Si are widely used in both rotating (i.e., motors and generators) and static (i.e., transformers) electrical machines (Fig. 1a). In static applications, as the magnetic flux is only required to travel in one direction, grain-oriented electrical steels are used to exploit anisotropy in the magnetic domain behaviour. In rotating applications, non-oriented electrical steels (NOES) are used to minimise the effects of magnetocrystalline anisotropy in a bulk specimen by avoiding any strong texture components of the microstructure.

Electrical steels are used due to their relatively high permeability and low coercivity, enabling the rapid magnetisation and demagnetisation required in rotating electrical machines^5^. This allows for high values of magnetic saturation, the mechanism of which depends on the spontaneous alignment of all the magnetic domains over the entire microstructure when applying an external magnetic field. Factors which inhibit the material from reaching saturation under an applied field may be referred to as hysteresis losses (Fig. 1c), leading to an increased energy demand to account for the inefficiencies.

It is common practice to evaluate the performance of NOES, and inherently the energy losses, by their magnetisation curves (Fig. 1c)^6^ (also referred to as **B***H* curves where **B** is the induced magnetisation and *H* is the applied magnetic field strength). Nevertheless, these performances are not only linked with the composition of the tested material^7^ but also how the magnetic domains are aligned with the crystallographic ‘easy’ axis (the preferential axis which is less energetically demanding for the magnetic flux to traverse, e.g., < 100 > for body centred cubic (BCC) Fe within their constituent grains^8,9^). Studies on magnetic domains have been carried out at the microstructural level historically with Kerr microscopy^10^, and more recently by forward scatter detector (FSD) imaging using an electron backscatter diffraction (EBSD) camera^11^. While these more in-depth analyses have been done on as-supplied NOES (i.e., after rolling), they do not explain the intimate relationship between the follow-up manufacturing methods (Fig. 1b) of raw material to become components in electrical motors – processes which inherently are likely to induce further losses^12^.

Therefore, without deep knowledge of material manufacture, the understanding of the captured magnetic domain observations is hindered. We see the effects of the convoluted magnetic domain textures, but we cannot fully understand the causes of these disturbances, which will impair the development of more efficient electrical machines.

The perturbances in the magnetic domain textures within a studied material or component can arise from various sources, e.g., preferential heat dissipation, crystallographic texture, possible inclusions, and mechanical loading^13^. As most of the production steps to manufacture electrical machines involve mechanical loading at different strain rates (e.g., cutting by various methods – punching, assembly, handling)^14–16^ there is a need to explore and understand the core phenomena on how the associated plastic deformations can influence the magnetic domains at the microstructural level (e.g., single grain) - Fig. 1d. Prior work has also demonstrated the influence of plastic strain on the magnetic domain texture of nanocrystalline Ni thin films through Kerr microscopy and magnetic force microscopy^17^, yet the strain-rate sensitivity of the magnetic response within a single crystal orientation should also be investigated.

Considering the above, it could be the case that even unexpected steps occurring in transient operations (e.g., pick-and-place) during the manufacture of electrical machines, could affect the micromagnetic structure of NOES.

A method to address these scenarios is to utilise micromechanical test methods (e.g., nano-indentation and micro-pillar compression) to understand the strain-rate sensitivity of a NOES, with an additional focus on the magnetic domain response. Whilst B-H testing is an acceptable method of characterising the magnetic behaviour of NOES after processing, from the practical perspective of an electrical machine designer, this macro-level approach does little to explain the sources of variation in magnetic performances when employing different manufacturing methods that often involve complex mechanical loading. As such, inferences can be made about what occurs to the magnetic domain texture at the micro level when (dynamic) mechanical loading is applied (e.g., impingement of garnet abrasive during abrasive waterjet machining^18,19^). However, in such cases, the material deformation is uncontrolled, so definitive statements regarding the effects of mechanically induced deformation upon the magnetic texture are difficult to interpret.

In this work, nano-indentation and micro-pillar compression, which are typically used to measure micromechanical properties in stress-affected areas^20,21^, are employed as a tool for inducing the stresses directly to investigate changes in the micromagnetic structure. This study of the relationship between the application of micro-loads and the corresponding magnetic domain disturbances gives insight into the governing mechanisms which lead to increased electrical losses in NOES (Fig. 1d).

## Results

### Application of controlled mechanical loading

Using a commercially available non-oriented electrical steel (Arnon 7 – FeSi 3 wt% Si), we induced mechanical deformation (see Fig. 1d) in single grains with their normal axes along the < 111 > direction using a nano-indenter with a spherical diamond tip, allowing us to investigate the fundamental effects caused by a single loading scenario on the magnetic domain structure. The < 111 > oriented grain was selected from an initial EBSD map prior to loading the sample in the nano-indenter assembly (see Supplementary Fig. 1). The < 111 > direction was chosen to increase the proportion of magnetic domains that are oriented out of the plane at the free-surface (||XY), therefore, increasing the magnetic signal measurement in the analyses of this surface. Whilst conventional nano-indentation is usually employed for mechanical property measurement at low strain rates^22^ to achieve stable, quasi-static loading, we were particularly interested in the time-dependent behaviour of the material at the micro-level and its effects on the magnetic domain texture; this is relevant for understanding the variations of macro-magnetic performances of NOES steels when processed by different methods. Further to nano-indentation with a spherical tip, where the bulk material surrounding the deformed region contributes to the system through an opposing force, we also consider the scenario where the deformation is isolated from the bulk material – through micro-pillar compression.

In this study, we selected two contrasting strain rates to apply the load as 10^−2^ s^−^^1^ and 10^1^ s^−^^1^, which we term LSR (low strain rate) and HSR (high strain rate), respectively, performing indents to a depth of 0.5 μm. The strain-rate values were chosen to encompass both quasi-static loading (LSR) and dynamic loading (HSR), whilst remaining within the data acquisition resolution of the nano-indenter load cell. The dynamic loading investigation has particular importance when considering the nature of the deformation events which may occur during material processing and operation (e.g., pick-and-place procedures, lamination stamping, debris impact) with their inherent effects on the magnetic domain texture.

### Magnetically affected zone around single deformation sites

**Fig. 2:**
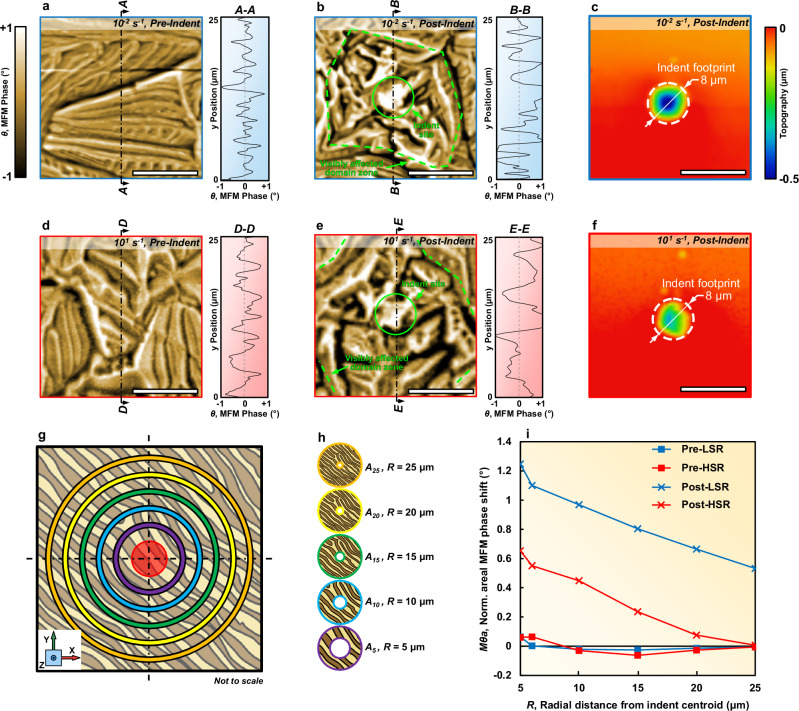
Magnetic domain disturbances caused by nano-indenter-induced deformation within single FeSi grains < 111 >. **a**, **d** Magnetic force microscopy (MFM) images of the sample free-surface (||XY) taken prior to indenting at low (10^−^^2^ s^−^^1^) and high (10^1^ s^−^^1^) strain rates, respectively, revealing the topographically independent base state of the magnetic domain structure. **b**, **e** MFM images taken post-indentation at low (10^−^^2^ s^−^^1^) and high (10^1^ s^−^^1^) strain rates, respectively, in the same location as (**a**, **d**), respectively. Line scans of the MFM Phase (*θ*) are shown adjacent to the maps (**a**, **b**, **d**, **e**). **c**, **f** Atomic force microscopy (AFM) topography maps of the surfaces shown in (**b**, **e**), respectively, post-indentation. **g**, **h** Schematic representation of the annular regions extracted from the MFM images taken before and after indentation, used for calculating the mean areal MFM phase shift in (**i**). The coloured rings in (**g**) correspond with the extracted areas in (**h**) of the same colours. The central red circle in (**g**) represents the indentation footprint, which is extracted from the calculation of the annular regions in (**h**). **i** Plots of the mean areal MFM phase shift (*Mθ*_*A*_) of the sample free-surface (||XY) before (square markers) and after (cross markers) indenting at low (10^−^^2^ s^−^^1^, blue lines) and high (10^1^ s^−^^1^, red lines) strain rates, revealing disparity in the magnetically disturbed areas – determined by the radial distance for the phase shift to reach the baseline (0°). The MFM colour bar applies to subfigures (**a**, **b**, **d**, **e**, **g**, **h**), indicating the centred MFM phase. The topography colour bar applies to sub-figures (**c**, **f)**, indicating the surface height as measured from the mean surface plane. The coordinate system shown in (**g**) is the same for all subfigures. Scale bars in all subfigures, 10 μm. Source data for plots are provided as Source Data files.

The investigatory procedure is detailed in Fig. 1d and the Methods section. It is already known that the complex behaviours of magnetic domains within a ferromagnetic material are related to magnetic anisotropies that can manifest in various forms (e.g., shape, magnetocrystalline, and stress-induced anisotropies)^23,24^. In our case, owing to the selection of a single unstrained grain (< 111 >), we can consider the magnetic anisotropy of a pristine, i.e., non-indented, zone to be minimal when referenced to the same individual grain. To verify this assumption, we used an atomic force microscope (AFM) with a magnetically polarised tip to perform magnetic force microscopy (MFM) within single grains. The selection used an initial EBSD inverse pole figure (IPF) map to identify the regions where indents were to be performed, and the pre-indentation regions were subsequently mapped using MFM (Fig. 2a, d) on the sample free-surface (||XY) (an example of the grain selection method through EBSD and MFM is provided in Supplementary Fig. 1). After that, we indented with the prescribed strain-rates in the pre-mapped regions and the MFM mapping procedure was repeated (Fig. 2b, e) (the loading curves for both LSR and HSR spherical nano-indentations are provided in the Supplementary Fig. 2). The MFM mapping pre- and post-indentation enabled the evaluation of the magnetic domain texture changes, which can be directly attributed to the application of quasi-static and dynamic mechanical loading. The MFM maps plot the centred phase shift (*θ*) in the probe oscillation measured during the lifted scan. The centred phase shift (after subtraction of the mean) is used instead of the absolute phase shift to account for any differences in the individual cantilever harmonics or tip magnetisation which may arise due to degradation of the consumable MFM tips. For both LSR and HSR indent locations, there is a visual difference in the magnetic domain structure before and after indentation – before, the MFM phase distribution shows less deviation from the mean in both the positive and negative directions (line scan insets of Fig. 2a, d). After indentation, the MFM phase distribution becomes more polarised – deviating from the mean and having greater magnitudes of phase shift between extremum (line scan insets of Fig. 2b, e), which indicates that there may be an obstruction to magnetisation through domain motion/growth, favouring the more, energetically demanding domain rotation^25^.

However, extracting further information visually about the magnetic domain textures from MFM has limitations. The dimensionality reduction which occurs when scanning the MFM probe through the surface magnetic field hinders the quantitative assessment of the complex patterns generated by the magnetic domains in the MFM image.

To address this, we propose a magneto-metrological procedure to measure the radial extent of the magnetically affected region surrounding the indentations. In Fig. 2g, h, we present a schematic representation of our procedure where we separated the MFM image into annular data subsets with regions, A_R_ of different radii (*R* = 5,…, 25 μm) centred on the indent footprint. The surface morphology contribution to the MFM map was controlled by using an interleave scan, where the topographic data was collected during the forward scan for both LSR and HSR surfaces (Fig. 2c, f respectively), enabling a constant lift height (100 nm) to be maintained between the tip and the surface during the lifted MFM scan (an animation visualising the forward and interleave scans is provided in Supplementary Fig. 3 and Supplementary Movie 1). The MFM phase data at the lift height can be considered valid for relatively flat samples, however, when larger features are encountered (i.e., an indent which could reach micrometre level depths) with non-planar surfaces, the tip encounters interactions from multiple directions, the components of which cannot be accounted for with the lift height alone – inducing artefacts in the MFM phase images. To account for this, when quantitively measuring the radial extent of the magnetic domain disorder, we did not consider the MFM phase values within the indent footprint (represented by the red circular area in Fig. 2g) – the circular area with a diameter of 8 µm from the indentation centre (shown in Fig. 2c, f). Therefore, we evaluated the changes in magnetic texture in the regions surrounding the indent footprint. For each A_R_, we determined the mean areal MFM phase shift of the surface *Mθ*_*A*_:

1 $${M\theta }_{A}=\frac{1}{{A}_{R}}{\mathop{\int \int}\limits_{{A}_{R}}}\,\left|\theta \left(x,y\right)\right|{\rm{d}}x{\rm{d}}y$$

where *A*_*R*_ is the area of the annular subset, and *θ* is the MFM phase at each position (*x*,*y*). By applying (1) to each of the annular subsets for both LSR and HSR regimes pre-, and post-indentation, we have revealed trends pertaining to the radial extent of the magnetically affected zone (Fig. 2i). The data for both LSR and HSR plots were normalised with respect to the mean *Mθ*_*A*_ value of their pre-indented surface, thus, enabling the comparison of the magnetically affected zones.

Firstly, as expected from initial visual observations, due to increased stress anisotropy, both LSR and HSR indentations elevated the *Mθ*_*A*_ relative to the baseline. However, the magnitude of the *Mθ*_*A*_ post-LSR was greater than that post-HSR, as was the radial distance required for *Mθ*_*A*_ to return to the baseline. In the HSR indentation, the indenter tip impacted the surface at a greater velocity than LSR, so one may assume that for indentations of a similar (0.5 μm) depth, increased indenter velocity would cause disturbances to the magnetic domain structure spanning a greater volume.

**Fig. 3:**
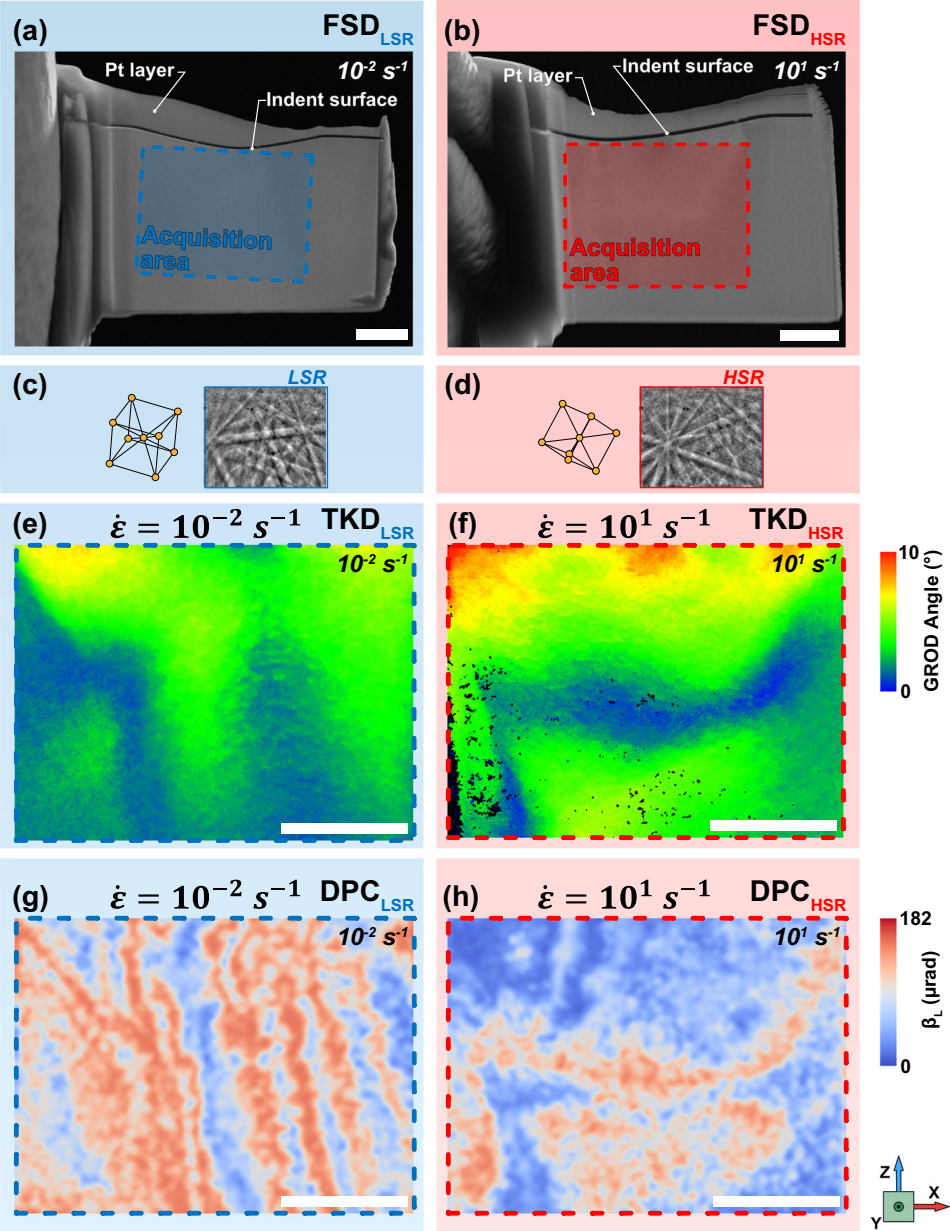
The effect of mechanically induced deformation on in-plane magnetic deflection within single grains. **a**, **b** FSD images of the extracted lamellae, cross-sectioning the indentations, made at low (0.01 s^−^^1^) and high (10.00 s^−^^1^) strain rates, respectively. **c**, **d** Extracted Kikuchi patterns and corresponding Fe-BCC unit cell representation from the cross-sectioned regions. **e**, **f** TKD GROD angle maps reveal sub-granular deformation by means of crystallographic misorientation. Map locations are the same as the acquisition areas annotated in (**a**, **b**), respectively. The colour bar for both (**e**, **f**) indicates the GROD angle at the same scale. **g**, **h** STEM DPC images showing the in-plane (||XZ) magnetic deflection in the same deformation regions as in (**e**, **f**), respectively, and an indication of strain-rate dependence of the magnetic domain structure. The colour bar for both (**g**, **h**) gives the magnitude of the in-plane (||XZ) magnetic deflection angle, β_L_, at the same scale. The coordinate system shown in (**a**) is the same for all subfigures (**a**, **b**, **e**, **f**, **g**, **h**). Scale bars in subfigures (**a**, **b**, **e**, **f**, **g**, **h**), 2 μm.

In HSR, the deformation occurs more rapidly than in LSR, limiting the formation and organisation of dislocation structures to the region directly below the indent^26–28^ (which we investigated later through cross-sectional studies in Fig. 3). The generated dislocations HSR lack the well-ordered pinning sites present in the case of LSR indentation, consequently, the interactions between abrupt dislocations directly below the indent and the magnetic domains are less pronounced on the surrounding free-surface (||XY). The limited pinning effect^29^ of the dislocations on the magnetic domain walls leads to relatively fewer distortions in the surface magnetic domain texture. On the other hand, it can be inferred that the lattice under LSR conditions has sufficient time to accommodate the deformation through dislocation movements. Therefore, in the LSR indentation, the gradual plastic deformation that is present can allow for the dislocations to be more organised than HSR, providing well-defined pinning sites for the magnetic domain walls to interact with. The strong interaction between the magnetic domain walls and the organised dislocations obstructs their movement away from a preferential axis (< 100 >) due to an increase in stress-anisotropy – presenting as alterations in the magnetic domain texture.

These findings highlight the role of time-dependent deformation in inducing localised changes in the magnetic domain texture on the sample-free surface (||XY). As such, the influence of ordered dislocations interfering with surface magnetic domains, leading to alterations in the material’s magnetic properties, is an interesting finding. It suggests that even quasi-static loading could have far-reaching effects on an electrical material’s magnetic behaviour. At a broader scope, similarly, low deformation rates may be encountered by soft magnetic materials during commercial usage (e.g., motor lamination assembly and centripetal loads in service), leading to potential difficulties in predicting magnetic performance.

To further investigate these aspects, it is imperative to analyse the material beneath the indentation (||XZ) to establish the relationship between the depth and magnitude of induced plastic deformation influenced by changes in strain rate, and the resultant magnetic domain texture.

### Magnetically affected zone beneath single deformation sites

To observe the deformation beneath the indents, lamellae cross-sectioning (in the ||XZ plane) both LSR and HSR indents from single grains of the same crystallographic orientation were extracted using the lift-out method within a focussed ion beam scanning electron microscope (FIB-SEM) and welded to copper grids for transmission analyses (Fig. 3a, b respectively, further details on the spherical nano-indentation cross-sectioning procedure are provided in Supplementary Fig. 6).

Through transmission Kikuchi diffraction (TKD) grain-referenced orientation deviation (GROD) mapping, we observed that the greatest magnitudes of deformation occurred directly below the indentation in both LSR (Fig. 3e) and HSR (Fig. 3f) regimes. The deformation zones beneath both indents also show defined contours between the areas of lower and higher misorientation. Under the LSR indent, these contours are more aligned with the loading direction (||Z) (Fig. 3e), whereas in HSR, there is a contour which traverses the lamella (||X) (Fig. 3f).

To understand the effects that mechanically induced deformation at low and high strain rates has on the magnetic texture, we performed scanning transmission electron microscopy – differential phase contrast (STEM-DPC) (Fig. 3g, h) with a pixellated detector to map the magnitude of the in-plane magnetic deflection, β_L_, beneath the indentations.

In LSR indentation, plastic deformation occurs primarily along the primary slip systems, which are easily activated under quasi-static conditions^30^. As the grain was loaded in the < 111 > direction, the {110} family of slip planes (the primary slip planes in BCC crystals) were easily activated. The more vertically oriented bands observed in the DPC map (Fig. 3g) suggest a correlation between the direction of the deformation network and the arrangement of magnetic structures.

Conversely, in HSR indentation, the deformation occurs rapidly, allowing only a limited time for dislocations to move and rearrange. This results in an altered arrangement of dislocations compared to LSR indentation. While the Peierls stress, which determines the resistance to dislocation motion, remains constant (i.e., is independent of strain rate), the rapid nature of HSR indentation increases dislocation interactions and density – especially near the indentation surface, due to constrained mobility. This results in localised stress concentrations and limits the volume for plastic deformation to diffuse into, leading to directionally altered magnetic structures, as seen in the two-dimensional cross-section view (Fig. 3h). Despite the region immediately below the indentation surface having a greater magnitude of deformation than that which occurred deeper, the corresponding DPC map (Fig. 3h) highlights that there is a stronger relationship between the dislocation network’s direction and the magnetic alignment than with the deformation magnitude. The differences in deformation behaviour between HSR and LSR highlight the time-dependent nature of dislocation motion and interactions during rapid loading, significantly affecting both the mechanical and magnetic properties of the material.

Tying information on the magnetic domains in the cross-sectional (||XZ) observations presented in Fig. 3 with those at the free-surface (||XY), shown in Fig. 2, we can conclude that the strain rate of applied deformation has a significant, time-dependent effect on the ability of the material to accommodate stresses. This has a marked effect on the magnetic textures at the level of a single grain. Application of LSR loads was found to induce deformation, which primarily altered the magnetic domain texture at the free surface (||XY) – Fig. 2i (see Post-LSR line). This may be attributed to the orientation of the activated, primary slip system intersecting with the surface, allowing the domain pinning effects to be observed on the external surface. However, for HSR load application, the rapid deformation results in a different dislocation arrangement rather than the activation of additional slip systems as the temperature remains constant for both LSR and HSR indentation. In this case, the deformation boundaries, with which magnetic structures were observed to align, are situated deeper below the free-surface (||XY) (shown in the cross-section - Fig. 3h). This had the effect of an apparent reduction in *Mθ*_*A*_ on the free-surface (||XY), requiring cross-sectional observation to determine the arrangement of the magnetic structures – Fig. 2i (see Post-HSR line).

**Fig. 4:**
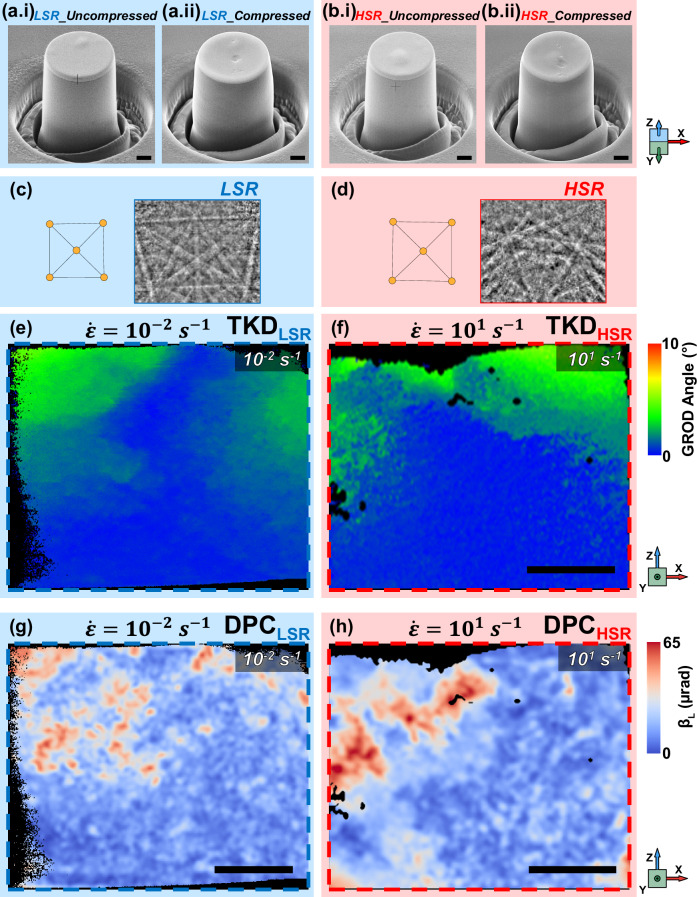
The effect of mechanically induced deformation on in-plane magnetic deflection within compressed pillars located in a single grain. **a**, **b** SEM images showing the pillars which were compressed at low (0.01 s^−^^1^) and at comparatively higher strain rates (10.00 s^−^^1^) before (**a.i**, **b.i**) and after (**a.ii**, **b.ii**) compression, respectively. **c**, **d** Extracted Kikuchi patterns and corresponding Fe-BCC unit cell representation from the cross-sectioned regions. **e**, **f** TKD GROD angle maps reveal sub-granular deformation by means of crystallographic misorientation. The lamellae for (**e**, **f**) were extracted by cross-sectioning (in the ||XZ plane) the compressed pillars shown in (**a.ii**, **b.ii**), respectively. The colour bar for both (**e**, **f**) indicates the GROD angle at the same scale. **g**, **h** STEM DPC images showing the in-plane (||XZ) magnetic deflection in the same compressed regions as in (**e**, **f**), respectively. The colour bar for both (**g**, **h**) gives the magnitude of the in-plane (||XZ) magnetic deflection angle, β_L_, at the same scale. The coordinate systems for the subfigures are given by the triad on the right of each row. Scale bars in all subfigures (**a**, **b**, **e**, **f**, **g**, **h**), 1 µm.

To provide more evidence for the observations discussed around the changes of magnetic domains within plastically deformed regions, we have performed further tests in different loading conditions. To simplify the loading scenario, and to avoid a complex stress distribution akin to that which occurred in the conventional spherical indentation (as above), we compressed a set of micro-pillars (Fig. 4a.i, b.i – pre-compression, Fig. 4a.ii, b.ii – post-compression at LSR and HSR respectively) which were located within a single grain oriented with the < 100 > direction of the crystal parallel to the defined X, Y, and Z axes (further details on grain identification and micro-pillar manufacture are provided in Supplementary Fig. 4). Compressing pillars ensures that the loaded area of the grain, hence the magnetic texture, is isolated from the neighbouring material. The pillars were deformed plastically to the same strain (*ε* = 0.078) at opposing strain rates. Like the spherical indents, we compressed the pillars plastically at both LSR (10^−^^2^ s^−^^1^) and HSR (10^1^ s^−^^1^) to isolate and provide more evidence of how the different deformation mechanisms can influence the distribution of magnetic textures. To further investigate how different deformation mechanisms influence magnetic textures, we conducted LSR and HSR compression tests on micro-pillars with the compression axis along the < 100 > direction. This orientation is different from the < 111 > loading direction used in the previously discussed spherical indentations. The change in crystal orientation allowed us to explore how the crystallographic direction of the applied stress affects the deformation and subsequent magnetic response. Due to this difference in orientation, we anticipated variations in the deformation patterns observed from TKD and the resulting magnetic alignment. This approach helps isolate the effects of strain rate from those of crystal orientation, providing a clearer understanding of how deformation dynamics influence magnetic properties.

For both LSR (Fig. 4e) and HSR (Fig. 4f) pillar compression, the deformation observed in the TKD maps predominantly aligns with the primary slip planes along the < 111 > direction. The corresponding DPC map for LSR pillar compression (Fig. 4g) shows a weakly banded magnetic structure, indicating that plastic deformation accumulates and organises along the easily activated primary slip planes, as expected under quasi-static conditions. However, the rapid deformation in HSR pillar compression leads to a denser and more complex dislocation network (rather than the activation of secondary slip systems) near the surface. The limited time for dislocations to move and reorganise under HSR conditions results in disordered interactions along dislocations, which in turn create complicated stress fields. This increased dislocation density and complexity provide additional boundaries and pathways for magnetic alignment, as seen in (Fig. 4h).

## Discussion

As the trend for electrification in society increases, it is vital to have a thorough understanding into how the functional properties of soft magnetic materials (e.g., NOES) may be altered from material synthesis, through device manufacture, and to end usage.

We demonstrate that by mechanically inducing deformation at low (10^−^^2^ s^−^^1^) and high (10^1^ s^−^^1^) strain rates within a single grain, the magnetic domain textures can be significantly altered. These changes are primarily due to the time-dependent dislocation dynamics and the resulting stress fields, which introduce pinning sites that can hinder magnetic domain wall motion during subsequent magnetisation. Here, we studied these phenomena on a commercial grade of NOES, which is composed of FeSi 3 wt% Si (with a Fe-BCC crystalline structure) as an example.

We have demonstrated how, at low strain rates, plastic deformation can influence the magnetic domain texture at the free surface (||XY) of the grain yet has little effect below the deformed site (||XZ). This could be due to the activation of primary slip systems in quasi-static conditions, which allows plastic deformation to accumulate along the activated planes in an organised manner, as there is sufficient time for the dislocations to be mobile. In contrast, the deformation at higher strain rates is more rapid, which leads to a more complex dislocation network, resulting in increased dislocation density and interactions near the free surface (||XY). This creates additional boundaries and pathways for magnetic structures, affecting the alignment and behaviour of magnetic domains, therefore, there can be less visible preferential alignment in the 2D cross-section view.

Furthermore, our results illustrate how the direction of applied loading to the crystal structure leads to an orientation-dependent response when observing changes to the magnetic texture. In our study, this was achieved by loading parallel to the < 111 > and < 100 > directions for the bulk and bulk-isolated tests, respectively.

The techniques adopted in our study (MFM, TKD, 4D-STEM) show a route to explore the micro-level magnetic properties of soft magnetic, electrical steels when subjected to various strain rates mirroring real-life manufacturing and operational conditions, demonstrating practical relevance. As such, there is future scope for advanced electrical material analyses at the micro level to understand how mechanical loads could impact the efficiency of electrical steels.

## Methods

### Sample preparation and site selection for magnetic domain mapping of the non-indented, free-surface (||XY)

To understand the influence of indentation strain rate upon the magnetic domain texture, there is a need to start from a surface with minimal defects (e.g., scratches, surface roughness, and contamination). As such, prior to performing the indentations, a flat sample of a commercially available NOES (Arnon 7 – FeSi 3 wt% Si) was prepared by grinding and polishing with 0.04 µm colloidal silica.

A large-scale EBSD survey of the sample was completed to build a dataset containing the crystallographic orientation information of many grains; this is of importance as the indentations were performed in similarly oriented grains, i.e., < 111 >. See an example of large-area EBSD mapping before indentation in Supplementary Fig. 1.

MFM was used to investigate the magnetic domain texture of the free-surface (||XY) pre- and post-indentation. MFM is a modified mode of AFM where an oscillating probe (nominal frequency = 75 KHz) with a sharp (maximum tip radius = 50 nm), magnetised (coercivity = 400 Oe) tip scans the topography of the sample surface along a single line. This topography is then retraced by the oscillating probe at a prescribed lift height (100 nm) above the surface, and the phase shift of the oscillation along the same line is plotted. The phase shift occurs due to interactions with the probe and the stray magnetic field above the sample surface, which is dictated by the arrangement of the magnetic domains^31^. The sampling locations for MFM were chosen with the aid of an optical microscope installed on the MFM scan head and the mild etching effect, which reveals the Fe-BCC grain boundaries when polishing with colloidal silica. Large area (50 x 50 µm) MFM scans were captured with 10% overlap between frames to generate a stitched MFM dataset. By combining MFM and EBSD datasets (an example is shown in Supplementary Fig. 1), we were able to observe the magnetic domain texture relative to the crystallographic orientations at known positions on the sample, and grains were selected for the initial indentation.

Based on the EBSD dataset (inverse pole figure (IPF) for the Z-direction), the selected grains for the LSR and HSR indentations and subsequent further analyses have a < 111 > axis direction parallel with the sample normal (*Z*-axis), the knowledge of which allows for the indentations to be made along the same directions in comparable grains.

### Nano-indentations (bulk deformation of single grain site) at opposing strain rates vs. maps of magnetic domain texture

After the target grains were identified and mapped in the pre-indentation state with MFM, the influence of the mechanical deformation upon the changes to the magnetic domain texture within a single grain could be investigated.

A diamond spherical indenter (Synton-MDP) with a 20 µm tip radius was used with the Alemnis Standard Assembly nano-indenter in displacement control mode (depth = 0.5 µm). This enabled the full indentation footprint (~ 8 µm diameter due to the elastic recovery of the material and compliance in the indenter system) to be captured in both the free surface (||XY) and cross-section (||XZ) analyses (i.e., when preparing the lamellae for TEM analysis). Two contrasting strain rates were tested to span 3 orders of magnitude (from 10^−^^2^ s^−^^1^ to 10^1^ s^−^^1^) to understand the strain-rate dependence of the magnetic domain texture.

While the LSR and HSR indentations were made to the same depth, and therefore had comparable geometry, the effect of dynamic indentation would inevitably lead to variations in lattice deformation and misorientation. Therefore, the application of contrasting strain rates would cause the magnetic domains to distort through different mechanisms.

To control interference from environmental variables, the indentations were completed at a high vacuum inside the chamber of an FEI Quanta650-ESEM, and the drift in the piezo actuator was stabilised by maintaining the nano-indenter assembly at these conditions for over 12 h.

### Mapping of magnetic domain distortion induced by nano-deformation within a single grain: Free-surface (||XY)

Post-indentation, the MFM maps were repeated at the indent sites, enabling the analysis of how the magnetic domain texture becomes distorted through the introduction of deformation alone. The degree to which the magnetic domain distortion extends beyond the physical boundaries of the indentation was enabled by the superposition of the traditional AFM topography data (collected on a second data channel during the MFM scan) over the MFM data. The topography data was levelled using a least squares plane fit, sampling the entire scan area but omitting features above (e.g., indentation pile-up) and below (i.e., the indentation volume) the surface. The overlapping of AFM and MFM mapping facilitated the spatial interpretation of data.

### Micro-pillar compression (isolated from bulk deformation in a single grain) at opposing strain rates for mapping the magnetic domain texture

**Fig. 5:**
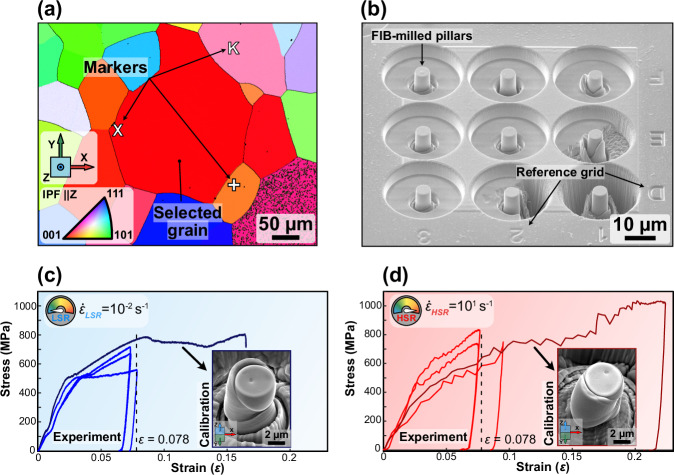
Site selection and preparation of micro-pillars to simplify the loading scenario. **a** EBSD inverse pole figure map showing the selected grain oriented //Z < 100 > (with associated stereographic key). **b** Example batch of micro-pillars during processing. Showing the FIB engraved coordinate system. **c**, **d** Stress-strain curves for the calibration pillars and the tested repetitions with inset images showing the calibration micro-pillars compressed to failure. Scale bars are located at the lower right of (**a**, **b**) and insets of (**c**, **d**). Source data for plots are provided as Source Data files.

To select the appropriate level of applied strain, we compressed two reference micro-pillars at low and high strain rates (LSR and HSR) to failure and extracted the stress-strain curves. For this calibration data to be relevant for the subsequent compressed pillars, we ensured that the crystallographic orientation of each pillar at a site within a single grain was identical by completing a large EBSD map prior to pillar milling (Supplementary Fig. 4a). A ZEISS Crossbeam 550 FIB-SEM fitted with an OINA Symmetry EBSD detector was used for this purpose. A large grain (area = 53,358 µm^2^ shown in Supplementary Fig. 4b.i) was identified for pillar milling (Fig. 5a) with orientation (< 100 > ||Z) so that it can accommodate many pillars while avoiding the errors that might be introduced by grain boundaries.

For this, markers (X, +, K) (annotated in Fig. 5a) were milled into the surface of the chosen grain to demarcate its boundaries so that the testing site could be located in subsequent operations. Two pads of Pt (75 μm x 75 μm) were deposited within the grain using the gas injection system (GIS) within the FIB to protect the material against Ga + implantation. A local reference grid (e.g., D, E, F and 1, 2, 3) was also milled adjacent to the Pt protective pads for pillar identification (Fig. 5b). The pillars were milled to a nominal diameter of 5 μm and height of 10 μm for use with the diamond indenter (6 μm diameter flat punch). The trenches surrounding the pillars were set to a diameter of 25 μm to aid in measuring the actual pillar heights and to provide probe access when removing the pillars later. For pillar milling, a roughing pass at 7 nA was performed, followed by two subsequent finishing passes of 1.5 nA and 0.3 nA. The FIB apertures were aligned to a 50 pA reference probe prior to the milling process to reduce exposure to higher FIB currents (i.e., reducing Ga + implantation) and to ensure concentricity between passes. The uncompressed, calibration micro-pillars were imaged at high resolution on a FEG-SEM to determine their diameter, height, and Pt layer thickness prior to indentation. The calibration pillars were compressed to failure at low and high strain rates to establish reference stress-strain curves (Fig. 5c, d, respectively). From the acquired curves, we selected the values of strain required to enter the plastic region ($\varepsilon \cong$ 0.078) (in situ videos (Supplementary Movies 2–5) show the micro-pillar compression and, are detailed in Supplementary Fig. 5). Following pillar compression, high-resolution FEG-SEM imaging was completed to identify failure mode, shearing direction and real plastic strain for the cases where the test was stopped prior to slipping/shearing activity. Using this calibration data, we compressed pillars located in a single grain to a level of strain prior to failure; this enabled the evaluation of changes in the magnetic texture after deformation at different strain rates.

### Analysis of the strain-rate sensitivity of the microstructure beneath both bulk and bulk-isolated deformation sites within single grain and the correlation with magnetic texture

The following investigatory method applies to both nano-indentations (bulk) and micro-pillars (bulk-isolated). As the strain rate was varied, residual plastic deformation and elastic recovery of different magnitudes occurred beneath the free surface (||XY), requiring the investigation of a cross-sectional view (||XZ) of the material under both the indentations and the compressed pillars.

Using the FEI Quanta200-ESEM, a protective Pt layer approximately 1 µm thick was deposited over the indentations and compressed pillars using the FIB. A low ion beam current of 50 pA was used to protect the free surface (||XY) against amorphization and ion implantation during the subsequent milling operations. The cross-sectional FIB milling was then completed at an ion beam current of 3 nA, the lamellae were lifted out, and Pt welded to a copper lift-out grid. Thinning of the lamellae was performed on the Zeiss Crossbeam 550 FIB-SEM to achieve a thickness of approximately 150 nm – enabling further transmission studies (further details regarding the lamella preparation method for both the spherical nano-indentations and the micro-pillars are provided in Supplementary Figs. 6 and 7, respectively).

To map the relative sub-granular deformation occurring beneath the indentations and compressed pillars, off-axis TKD was performed with an accelerating voltage of 30 kV, an electron beam current of 1.0 nA, and a pixel size of 0.02 µm. These parameters allowed us to obtain a high level of angular precision for mapping the grain-referenced orientation deviation (GROD) angle for each scan point in the crystal lattice beneath the indentations and compressed pillars. It should also be noted that the act of thinning the deformed regions (indentations and compressed pillars) into lamellae may cause some dislocation relaxation – which could lead to an apparent reduction in the dislocation density when measured with TKD. A solution to this problem has been proposed and explored successfully by Kalácska et al.^32^ where serial FIB slicing was used on deformed, square, copper micro-pillars to observe the dislocation network in 3D to obtain more reliable results on the geometrically necessary dislocation (GND) density. However, this would require the grain of interest to be positioned on the edge of the sample, and the method is destructive – limiting further analysis of the sample with STEM methods, placing this method outside the scope of the current study.

**Fig. 6:**
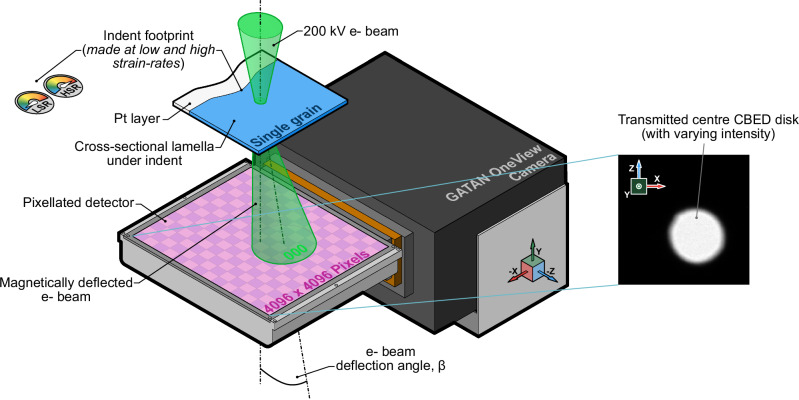
STEM differential phase contrast procedure with a pixellated detector. For isolating the magnetic components of contrast from the material deformation observed in TKD. An example of the central CBED disk is displayed on the pixellated detector.

Scanning transmission electron microscopy (STEM), was used for the isolation of contrast arising from magnetic origins. The STEM images were captured using a Gatan OneView pixelated detector within a JEOL 2100 + TEM at 200 kV (Fig. 6). A large camera length (150 cm) was used with a 10 µm aperture, giving a convergence semi-angle of 2.35 mrad, enabling the imaging of the central transmitted disk in the convergent beam electron diffraction (CBED) image. It should be noted that greater angular precision of the central disk could be achieved by using a larger aperture (e.g., using a 50 µm aperture would yield a convergence semi-angle of 13.2 mrad); however, this would limit the working distance of the electron beam causing the focus value to vary between the upper and lower faces of the sample. For both the LSR and HSR lamellae, scans measuring ~ 6 x 4 µm were made in the same locations as the TKD maps.

To process the 4D-STEM^33^ data, the CBED images were extracted from the native.dm4 files using the HyperSpy library^34^. It should be noted that file size becomes a significant constraint with 4D-STEM datasets, so the detector may be binned at the data collection stage to ensure that the dataset is not too large for practical post-processing. To reduce the dataset size further, we computed an average CBED image from the full scan area and square-cropped all of the CBED images to the central disk only. To correct for the influence of scan position on the central disk location as the electron beam is translated over the sample, we applied a Sobel operator to emphasise the disk edges, fitting a circle to the edge image using Taubin’s singular value decomposition (SVD) method^35^. An affine transformation was applied to the individual CBED images to position the central disk in the centre of the cropped region. Following the scan correction procedure, we calculated the centre-of-mass (CoM) of the central spot intensity, producing a map of beam displacements after interaction with the sample. These displacements are caused by interactions with the magnetic domains within the material and the electron beam due to the Lorentz force, with the deflection angle, *β*_*L*_, relating to magnetic induction, **B**_**S**_, by:

2 $${\beta }_{L}=-\frac{e\lambda }{h}{{{\bf{B}}}}_{{{\bf{s}}}}t,$$

where *e* is the magnitude of electron charge, *λ* is the relativistic electron wavelength at an accelerating voltage of 200 kV, *t* is the sample thickness, and *h* is Planck’s constant^36,37^.

Mapping *β*_*L*_ in the same region as the TKD maps using (2) has enabled the correlative analysis of sub-surface deformation structures with magnetic textures, revealing the strain-rate dependency of their relationship.

## Supplementary information

Supplementary Information

Description of Additional Supplementary Files

Supplementary Movie 1

Supplementary Movie 2

Supplementary Movie 3

Supplementary Movie 4

Supplementary Movie 5

## Source data

Source Data

Transparent Peer Review file

## Data Availability

The datasets generated during and/or analysed during the current study are available from the corresponding author on request. Source data are provided in this paper.
