# Supplementary material for: Effect of sub-micron deformations at opposing strain rates on the micromagnetic behaviour of non-oriented electrical steel
Source: Nat Commun. 2024 Oct 18;15:9010. doi: 10.1038/s41467-024-53346-7 (PMC11489813; doi:10.1038/s41467-024-53346-7)
Supplement: Supplementary file 1 — Supplementary Information [file 41467_2024_53346_MOESM1_ESM.pdf]

Supplementary Information

Manuscript Title:

Effect of sub-micron deformations at opposing strain rates on the micromagnetic behaviour of non-oriented electrical steel

Author List:

Kieran Winter[1], Zhirong Liao[1]*, Erik Abbá[1], Jose A. Robles Linares[1], Dragos Axinte[1]**

Corresponding Authors:

* Zhirong.Liao@Nottingham.ac.uk

** Dragos.Axinte@Nottingham.ac.uk

Affiliations:

[1] Rolls-Royce University Technology Centre in Manufacturing and On-Wing Technology, Faculty of Engineering, University of Nottingham, UK

Supplementary Figure 1 – Example of grain selection method for nano-indentation in the ||XY plane

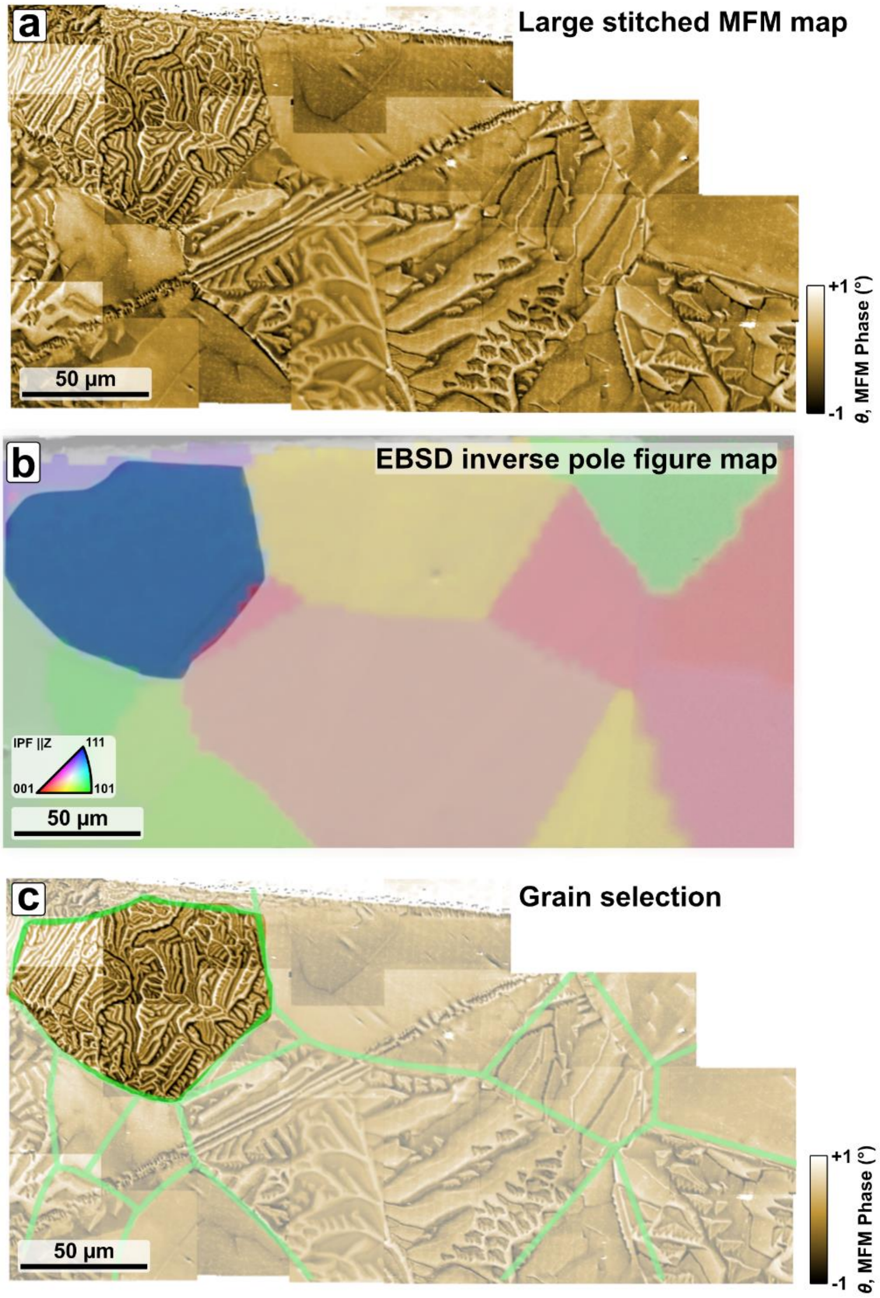

Supplementary Figure 1 Nano-indentation grain selection procedure. a Large, stitched MFM map showing the MFM Phase, θ, on the sample free-surface ||XY. **b** EBSD inverse pole figure (IPF) map of the same region mapped in **a**. The selected grain is highlighted and the colour corresponds to the stereographic triangle in the inset key. **c** Large, stitched MFM map as shown in **a**, with the grain boundaries measured in **b** superimposed to highlight the chosen grain. Colour scale for subfigures **a, c**, are located to the right of the respective maps. Scalebars for subfigures **a-c**, are located at the lower left of each map.

Supplementary Figure 2 – Loading curves for spherical nano-indentations at low and high strain-rates

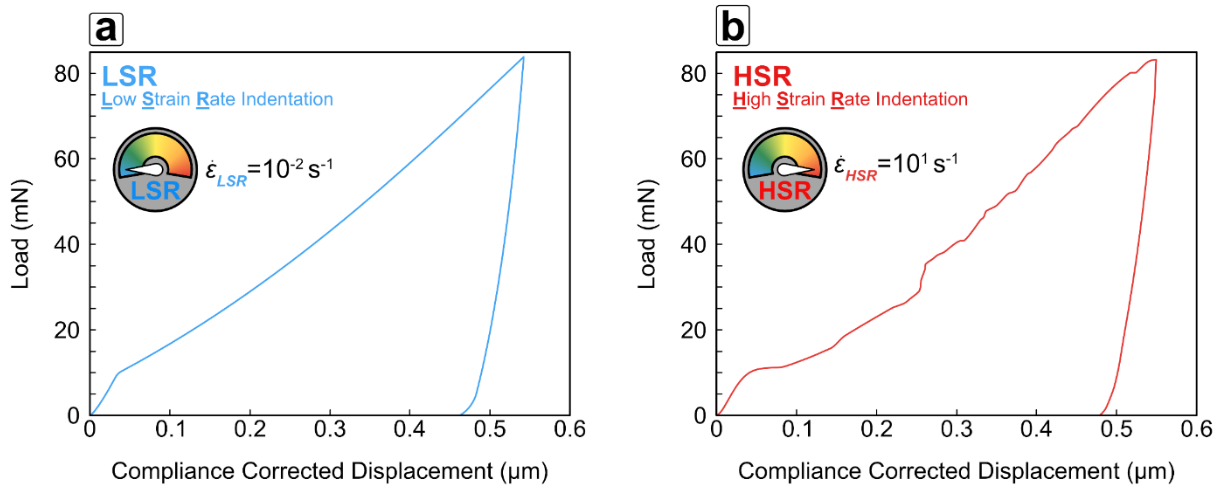

Supplementary Figure 2 Loading curves for spherical nano-indentations. The loading curves are shown for both the low (LSR) and high (HSR) strain rate nano-indentations in **a, b,** respectively.

Supplementary Figure 3 – MFM scan procedure - Video

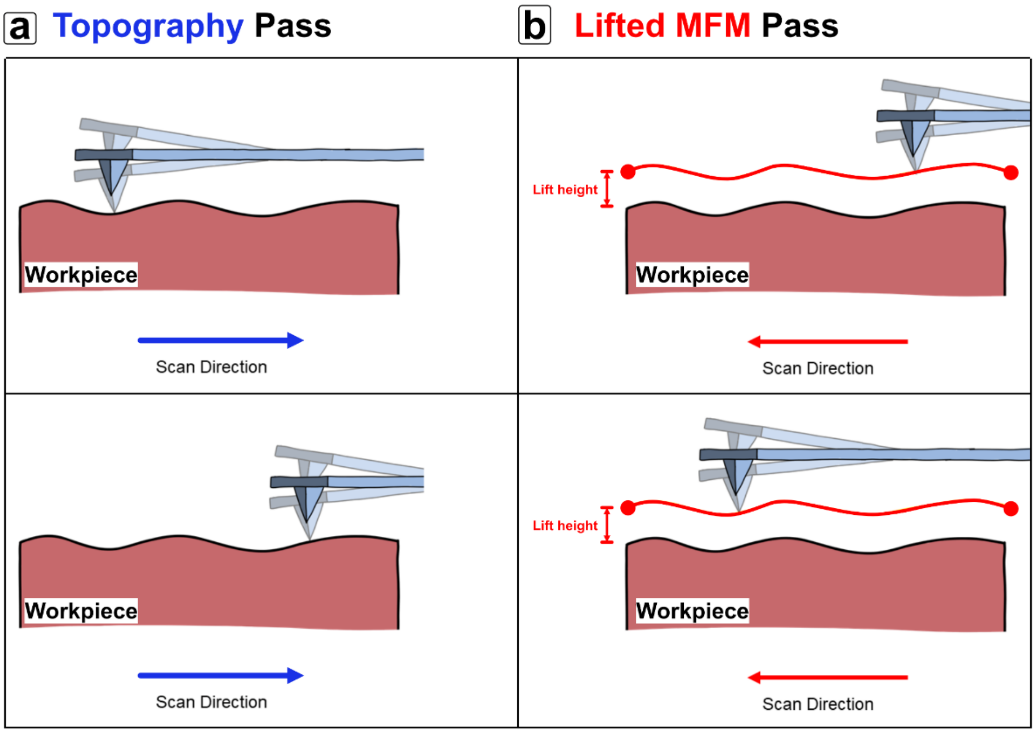

Supplementary Figure 3 MFM mapping procedure. Frames from the supplementary animation of the MFM mapping procedure. Column **a** depicts the forward scan where the surface topography is mapped. Column **b** depicts the lifted scan where the cantilever and tip is raised to a set height above the surface measured in the forward scan, **a**. Note: Video 1 shows animated MFM scanning procedure.

Supplementary Figure 4 – Expanded micro-pillar procedure: Locating and FIB milling.

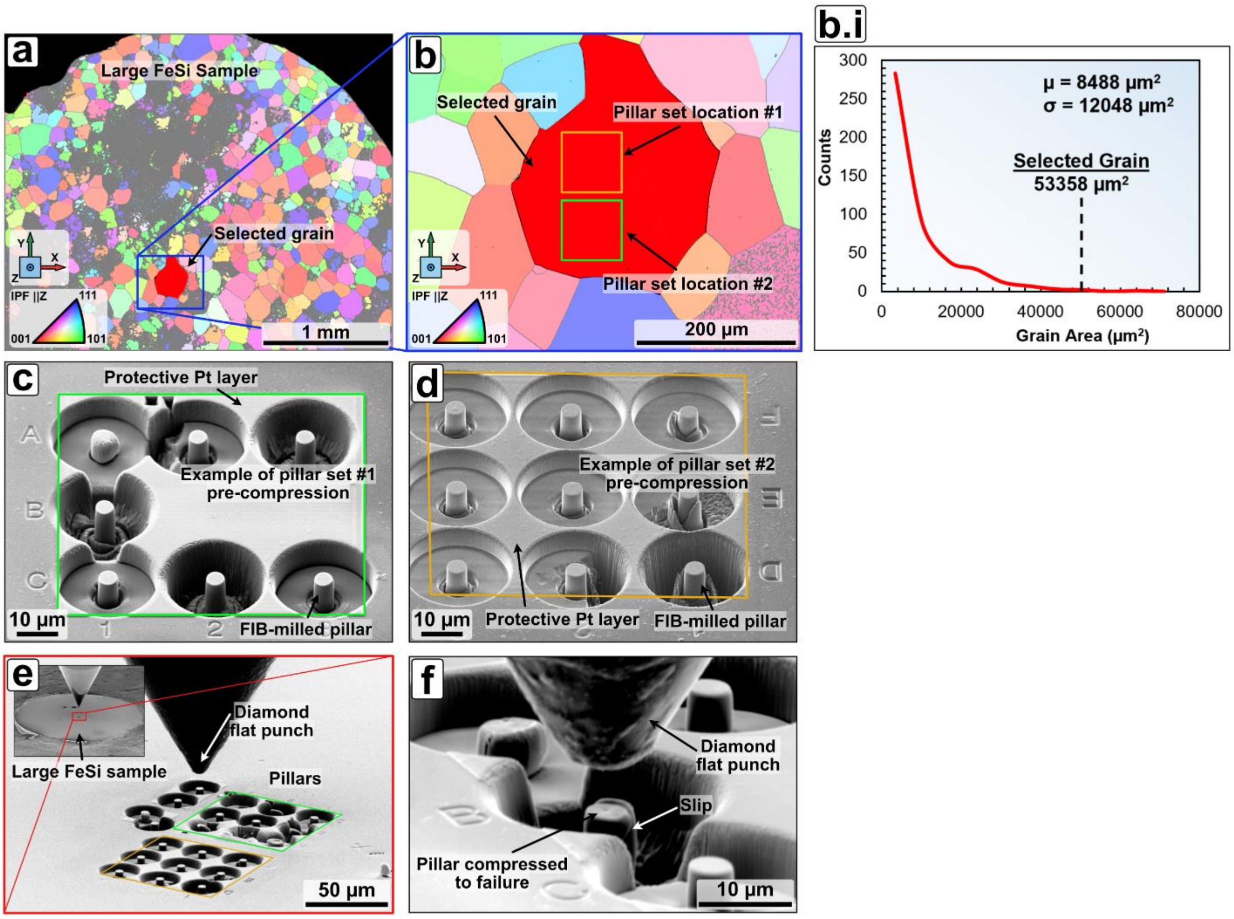

Supplementary Figure 4 Expanded micro-pillar procedure: Locating and FIB milling. a Preliminary, coarse EBSD inverse pole figure map used to identify potential grain candidates. **b** Increased resolution EBSD inverse pole figure map with the selected grain highlighted. **b.i,** Grain size statistical plot with the area of the selected grain from **b** marked with a dotted line. **c, d,** Regions where micro-pillars were manufactured within green and orange squares located in the single grain shown in **b**, respectively. **e** Overview in-situ SEM image showing the diamond flat punch positioned over the micro-pillars pre-compression. **f** SEM image taken post-compression of a micro-pillar which has been compressed to failure showing slip.

Supplementary Figure 5 – Micro-pillar compression testing

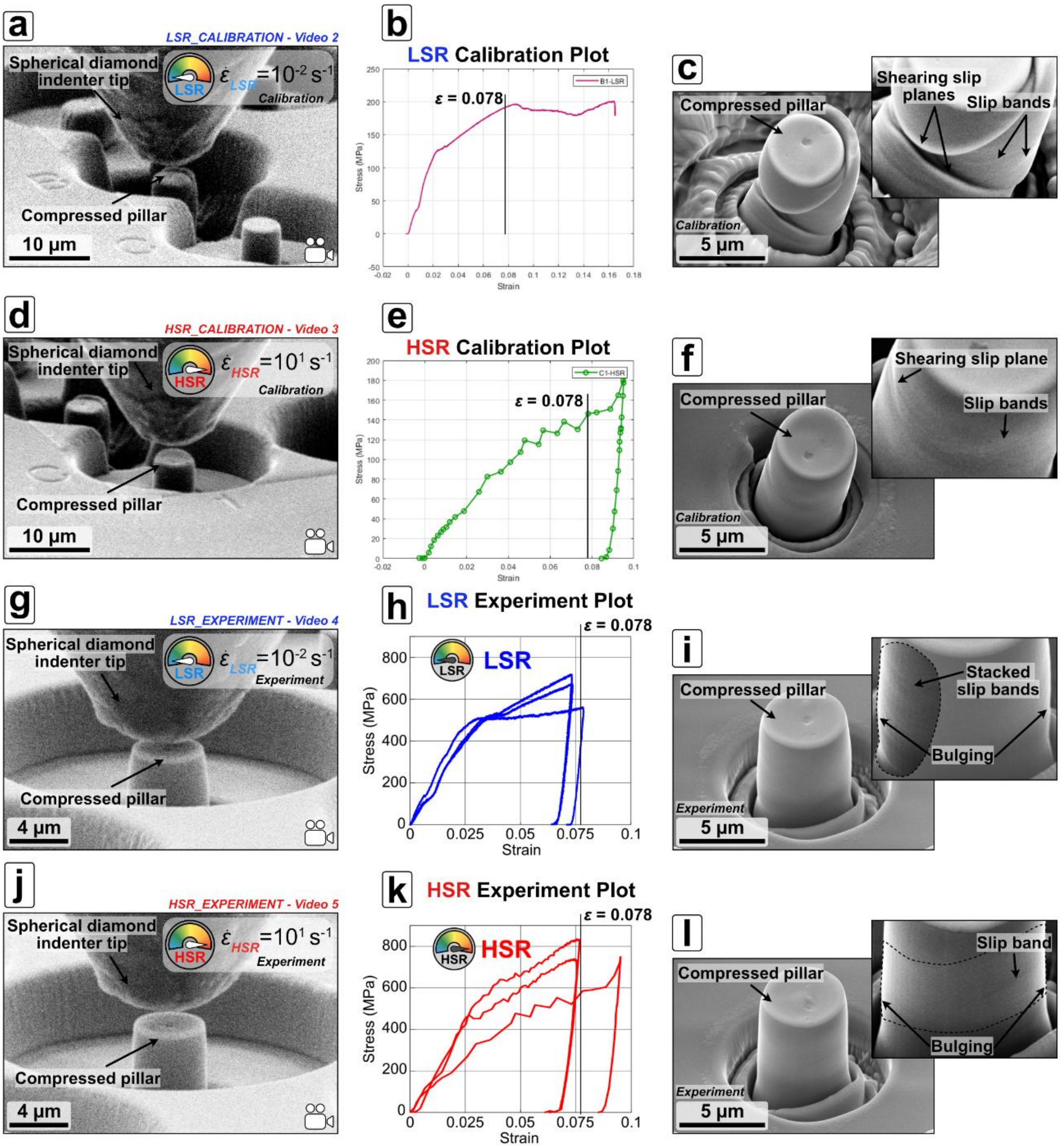

Supplementary Figure 5 Micro-pillar compression. a, d, g, j In-situ SEM video frames after micro-pillar compression. **b, e, h, k** Stress-strain curves measured during micro-pillar compression tests. **c, f, i, l** FEG-SEM images of the compressed micro-pillars with inset magnifications highlighting the deformation.

Note: Subfigures in the rows **a-c** and **d-f** are extracted from the calibration tests where micro-pillars were compressed to failure at low and high strain rates respectively. Subfigures in the rows **g-i** and **j-l** are extracted from the experiment tests where micro-pillars were compressed to a strain (ε=0.078) selected with aid from the calibration curves (**b** and **e**) at low and high strain rates respectively. The in-situ SEM videos (Video 2 – 5) of the micro-pillar compression shown in **a, d, g, j** are attached.

Supplementary Figure 6 – Cross-sectioning procedure for analysing the spherical nano-indentation

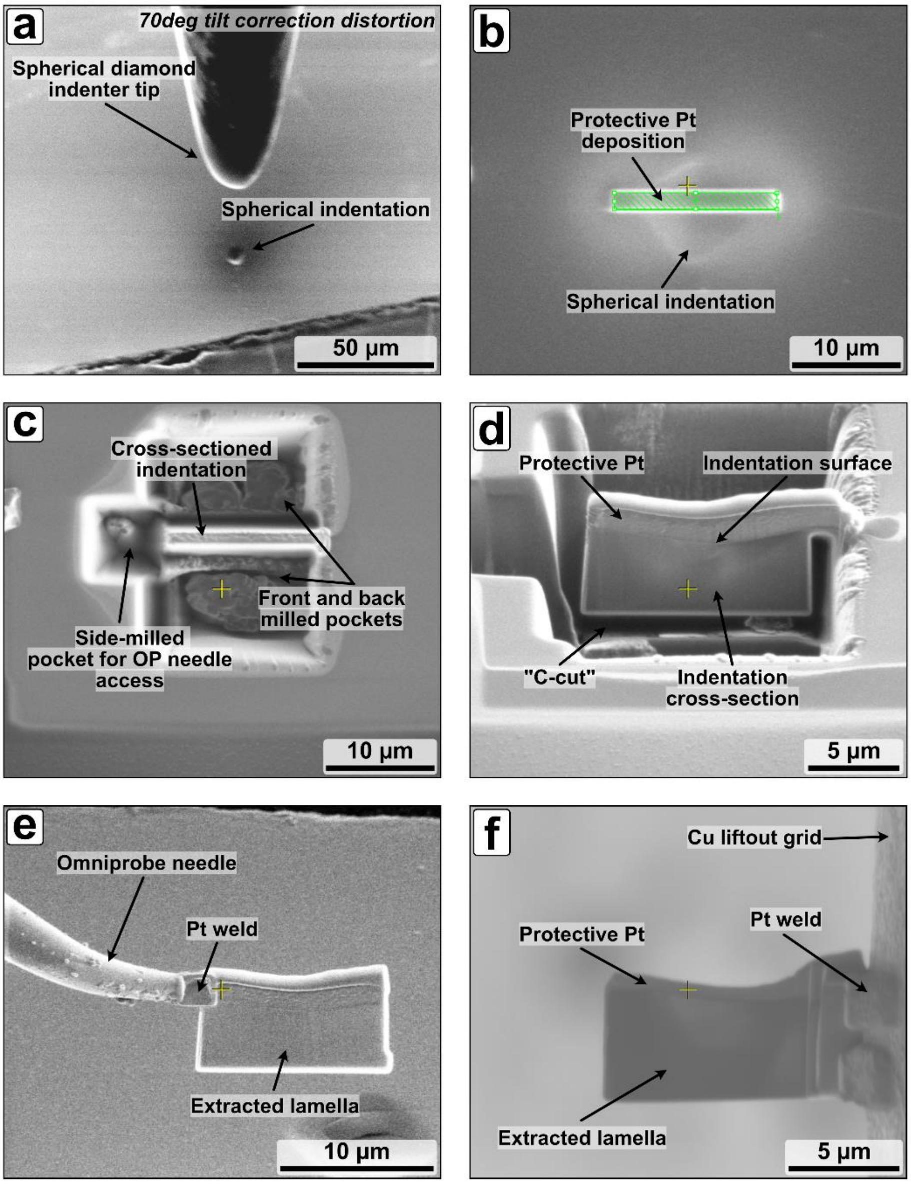

Supplementary Figure 6 Cross-sectioning procedure for analysing the spherical nano-indentations. a In-situ SEM image showing the diamond spherical indenter tip (radius 20 µm) positioned above a spherical indentation. **b** FIB image taken when depositing a protective Pt cap over the spherical indentation to protect it from Ga+ damage during subsequent milling operations. **c** FIB image showing the front and back milled pockets used for cross-sectioning the spherical indentation and the side-milled pocket to allow for OmniProbe needle access for lamella extraction. **d** FIB image showing the lamella pre-extraction after being released from the bulk material with a "*C-cut*". **e** FIB image of the extracted lamella being lifted out of the bulk material after Pt welding to the OmniProbe needle. **f** Lamella taken from the cross-section of a spherical nano-indentation Pt welded onto a Cu liftout grid ready for transmission studies (transmission Kikuchi diffraction and 4DSTEM).

Supplementary Figure 7 – Cross-sectioning procedure for compressed micro-pillars

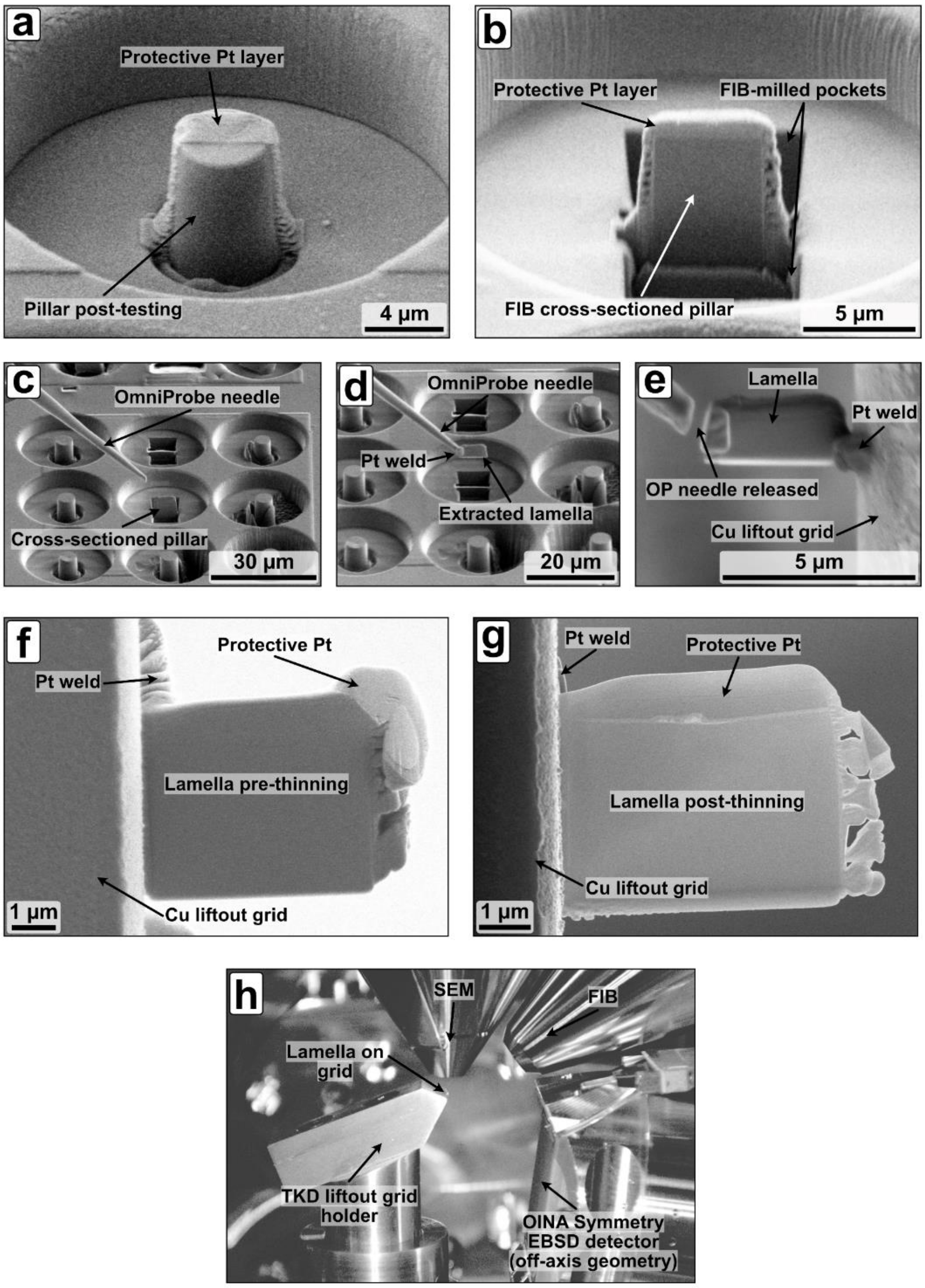

Supplementary Figure 7 Cross-sectioning procedure for compressed micro-pillars. a SEM image of a compressed micro-pillar with a secondary protective layer of Pt to prevent Ga+ damage in subsequent cross-sectional FIB milling. **b** SEM image of a cross-section of a compressed micro-pillar prior to extraction. **c** FIB image showing the OmniProbe needle positioned above a cross-sectioned micro-pillar. **d** FIB image of the lamella Pt welded onto the tip of the OmniProbe needle and extracted from the bulk material. **e** Extracted lamella Pt welded onto a Cu liftout grid showing the stage where the OmniProbe needle is released by FIB milling. **f, g** Extracted lamella on Cu liftout grid before and after FIB thinning, respectively. **h** SEM chamber camera image for the TKD experiments using off-axis detector geometry.
