# Supplementary material for: Effect of sub-micron deformations at opposing strain rates on the micromagnetic behaviour of non-oriented electrical steel
Source: Nat Commun. 2024 Oct 18;15:9010. doi: 10.1038/s41467-024-53346-7 (PMC11489813; doi:10.1038/s41467-024-53346-7)
Supplement: Supplementary file 2 — Description of Additional Supplementary Files [file 41467_2024_53346_MOESM2_ESM.pdf]

Description of Additional Supplementary Files

Supplementary Movie 1
Animation showing the magnetic force microscopy (MFM) scan procedure.

Supplementary Movie 2
In-situ video of the low strain-rate compression of a calibration micro-pillar.

Supplementary Movie 3
In-situ video of the high strain-rate compression of a calibration micro-pillar.

Supplementary Movie 4
In-situ video of the low strain-rate compression of an experiment micro-pillar.

Supplementary Movie 5
In-situ video of the high strain-rate compression of an experiment micro-pillar.
