# Supplementary material for: Effect of sub-micron deformations at opposing strain rates on the micromagnetic behaviour of non-oriented electrical steel
Source: Nat Commun. 2024 Oct 18;15:9010. doi: 10.1038/s41467-024-53346-7 (PMC11489813; doi:10.1038/s41467-024-53346-7)
Supplement: Supplementary file 8 — Source Data [file 41467_2024_53346_MOESM8_ESM.pdf]

nature portfolio

Peer Review File

Effect of sub-micron deformations at opposing strain rates on the micromagnetic behaviour of non-oriented electrical steel

Corresponding Author: Dr Zhirong Liao

This file contains all reviewer reports in order by version, followed by all author rebuttals in order by version.

Version 0:

Reviewer comments:

Reviewer #1

(Remarks to the Author)
I found the article acceptable.

Reviewer #2

(Remarks to the Author)
The manuscript presents study of the influence of applied strain on micro-level magnetic properties for non-oriented electrical steels with magnetic force microscopy, transmission Kikuchi diffraction and 4D Scanning transmission electron microscopy. Magnetic structure behaviour was investigated within a single grain.
I find this manuscript well written and presented. The quality of data is good and it was obtained with appropriate experimental techniques and thoroughly analyzed. For example, authors used on-axis transmission Kikuchi diffraction geometry with horizontal screen that excludes the distortions (with increased spatial resolution in comparison to off-axis TKD, EBSD). TKD is a promising new technique for the analysis and routine characterisation of nanocrystalline materials and nanostructures, capable of effective resolutions of down to few nanometres.
Novelty, motivation of the research and provided results are clear and well explained. Especially, I find Figure 2 very helpful for the reader because it makes clear what information was obtained with different experimental techniques and explains the structure of experimental part of the manuscript.
The authors convinced me that those results have potential significance for the field of manufacturing of the electrical machines because, procedures that are applied during production can influence the magnetic domains at the microstructural level and should be taken into account.
I do not have any doubts about the validity of the approaches, that were used for investigation, and the quality of the obtained data, but I would like to ask the authors to provide more clear evidence of their claims. Namely:
-In the text before Fig. 5 authors state “A possible explanation for the load jump (for HSR) is that strain was transferred deeper into the bulk material, inducing plastic deformation before dislocations were able to accumulate in the region directly under the primary deformation zone (Figure 5f-iii). This indicates that the region with a low misorientation angle (Figure 5d, Figure 5f-iv) is only deformed elastically when transmitting the strain to the bulk material.“
It would be nice if authors explained this statement in more details: it is unclear to me why in case of HSR strain was transferred deeper including plastic deformations but on the other hand the region with small misorientation angle is ONLY deformed elastically. It is even more confusing later when authors claim ”The contour beneath the primary zone which is surrounded by plastic deformation (Figure 5f-iv), shows a relative increase in magnetisation, with a demagnetisation band observed directly below, at the boundary (Figure 5f-v) with the secondary deformation zone (Figure 5f-vi)”. Because, in the Figure 5f-iv it is written that region iv is “Elastic transfer zone”.
-In the abstract authors write “… we reveal that low velocity indentations result in magnetic deflections away from the deformed zone when compared with the higher velocity which has a higher degree of alignment due to defect-pinning“. But if one compares Figure 5 (g and h) for LSR and HSR respectively, it could be seen that magnetic deflection appears at the same coordinates for Z and X. They maybe oriented parallel to X in case of HSR and parallel to Z in case of LSR but, still one can find red regions (that correspond to high β_L) across the whole acquisition area. Furthermore, in Figure 5 (h) there are no areas with high β_L close to indent surface in contrary to Figure 5 (g) that corresponds to LSR. Please, clarify this in more details.

I have some suggestions that from my point of view could improve the manuscript:
-Please, state the spatial resolution for TKD measurements. I assume, that with the thickness of the lamellae about 150nm, accelerating voltage of 30kV and electron beam current of 1.0 nA, spatial resolution is about 20nm or higher?
-It would be interesting to see Kikuchi patterns for the selected single grain for LSR and HSR at least in the Supplementary materials. Are there any differences between them?
-Does colour-code for Figure 5 (c-d) correspond to the colour-code of the stereographic triangle in Figure 2? Please clarify this in the caption for Figure 5.
-Maybe one can add to supplementary material a plot that represents statistics of the EBSD-determined grain sizes?
-Please, check one more time notations in the text (numbers and letters for figures). There are my suggestions:
Line 240: the abbreviation “TKD GROD” appears in the capture of Figure 5 earlier than it is explained in the text;
Line 244: colour bar for (g) and (h)?
Line 251: For HSR (Figure 5 h)?
Line 265: Tying the cross-sectional (||XZ) observations presented in Figure 5 ?
Line 266: free-surface (||XY), shown in Figures 3 and 4?

Reviewer #3

(Remarks to the Author)
The manuscript reports on a study on the effect of mechanical deformations on microscale magnetic behavior of non-oriented electrical steel. The main strength of the work is undoubtedly represented by the synergistic approach which takes advantage of the use of different techniques allowing the selection of single grains, their indentation, the magnetic mapping, and the cross sectional analysis under the indented area.
Despite this interesting approach, the manuscript does not contains a clear description of the results and a comprehensive rationalization of the results. Also, although the results can be interesting, they do not seem to justify their publication in Nature Communications. I therefore would recommend the rejection of the manuscript, although a resubmission can be considered after a careful revision of the manuscript. Below my comments as I found them reading the manuscript.
- overall, the English writing a significant improvement. Also the structure of some sentences seems broken and should be revised.
- the introduction starts with a figure which is decorative but without a practical importance for the manuscript, followed by a sort of graphical abstract describing the scope of the work. This is definitely confusing and should be rationalized.
- the introduction end with the sentence “Not to be regarded as a scope of the study but…” which adds a further element of confusion. It would be better deleted or at least moved in the discussion.
- the “Results” section is actually a not so rational merge of experimental section, with materials and methods, and results. The structure should be rationalized, for instance reporting what is actually a “materials and methods” topic in the next session.
- the authors asses they selected the <111> direction grain. How was this done? Do they identified the grain “outside” the nanoindenter and then moved the sample or was it “in situ”? Please detail. I assume the procedure is the one described later about MFM, but please clarify.
- line 130: “The details of the investigatory procedure are detailed in Figure 2 and the methods.” please fix this sentence.
- line 144: “before the MFM phase distribution shows a gradient between opposing polarities”: what does it means? Are the authors referring to a smooth variation? Maybe some profiles could help.
- together with MFM images, topography images of the surface should be added. In particular because the topography may often induce electrostatic artifacts in MFM phase images and thus the possible morphology contribution to the MFM map should be discussed.
- The authors report the roughness measured as to be 0.447 ± 0.0725 nm. It is not clear the importance of this parameter in the work. Also, if the information is maintained, please rationalize the significant digits of the mean and the uncertainty.
- the method for the analysis of the radial extent of the magnetic is interesting. However, some clarifications are needed, e.g., “The indent footprint itself was also removed from the annular subsets to avoid the inclusion of MFM…” what does it mean?
- the method requires the integration over an circular region. Is the anisotropy relevant for this method? I mean, a large scale anisotropy due to the different grain orientation…
- it is not clear which phase shift the authors use: is it the absolute shift or is the phase after subtraction of the mean (as it seems from Fig. 3)?
- in supplementary materials, please note that MFM images are referred to as AFM.

Reviewer #4

(Remarks to the Author)
This manuscript aims to demonstrate that deformation resulted from indentation at different stain rates affects the magnetic domain distribution. Characterization techniques such as MFM, TKD and STEM/DPC were used. The re-arrangement of magnetic domains in a small area under stress/strain application has long been demonstrated (for example, Zhang et al., Nuclear Instruments and Methods in Physics Research B, 2007, 257, 379-382). This study, however, failed to further correlate plastic strain and domain re-arrangement. For example, high GROD values for the HSR sample are observed near the surface as compared to those for the LSR one, whereas higher MFM phase shifts are found for the latter. Moreover, the high GROD values mentioned above should result in a higher rotation of the magnetic moments. The projection of the saturated magnetization (Bs) on the sample surface and resulted change of magnetic deflection angle (beta L) should vary from one position to another on surface. However, the DPC map does not correspond to the argument. Accordingly, this manuscript shows preliminary attempts to analyze local changes of magnetic domain structure on strain, but more efforts are

needed to quantify the results.

Version 1:

Reviewer comments:

Reviewer #3

(Remarks to the Author)
I thank the Authors for how they addressed the comments. I think the manuscript can be published as it is.

Reviewer #5

(Remarks to the Author)
The micro-pillar work was greatly appreciated. It greatly reduce the stress complexity as compared to spherical indentation tests therefore the effects of crystal orientation and strain-rate can be discussed more clearly.

Just a few more questions and comments on how magnetic deflection relates to micro-deformation:
1. In Fig. 4(c) and (d), the Kikuchi patterns of the lamellar show different orientation. What is the explanation for this if the lamellar were all FIBBed from the center of the pillar?

2. The author used the concept that HSR tests provide higher energy hence able to activate more slip systems; I found this somehow misleading and need to be corrected. Plasticity in BCC metals are thermally-activated and strain-rate dependent, e.g. BCC metals exhibit higher plasticity at elevated temperatures and lower strain-rate. The thermally-activated dislocations are carrier of plasticity, if provided with sufficient time. This can be seen in GROD maps from HSR tests in Fig. 3(f) and Fig. 4(f), where higher amount of deformations (higher GROD value) accumulated at the surface since mobile dislocations lack sufficient time to move outwards, relieving the accumulated stresses. Just to be clear, the Peierls stress for the dislocation motion on the activated slip system are the same for LSR and HSR tests, therefore HSR tests will NOT activate additional slip systems since crystal orientation and temperature of the two test scenario are the same

3. The usage of TKD techniques can also be quite misleading, as the stress-state are far from the as-compressed state due to the thinning. Can the author provide non-TKD configuration (clean pillar surface using FIB) for GROD calculation? There are some very nice work done by Kalacska et al. on characterizing micro-deformations of Cu micro-pillars (MSEA, 770(7), 2020). Her work was also done in conventional EBSD mode so should be a nice reference for calculation the deformation underneath the Fe-3Si micro-pillar.

This is excellent work so I'd appreciate using the suitable dislocation-based mechanics to explain plasticity, and additional work for characterizing the micro-deformations using EBSD technique.

Version 2:

Reviewer comments:

Reviewer #5

(Remarks to the Author)
I am satisfied with the author's responses of my comments. I think the manuscript can be published as it is now.

Open Access This Peer Review File is licensed under a Creative Commons Attribution 4.0 International License, which permits use, sharing, adaptation, distribution and reproduction in any medium or format, as long as you give appropriate credit to the original author(s) and the source, provide a link to the Creative Commons license, and indicate if changes were made.
In cases where reviewers are anonymous, credit should be given to 'Anonymous Referee' and the source.
The images or other third party material in this Peer Review File are included in the article's Creative Commons license, unless indicated otherwise in a credit line to the material. If material is not included in the article's Creative Commons license and your intended use is not permitted by statutory regulation or exceeds the permitted use, you will need to obtain permission directly from the copyright holder.
To view a copy of this license, visit https://creativecommons.org/licenses/by/4.0/

The authors would like to thank the reviewers for their valuable feedback to improve the quality of our submission.

Reviewer Comments:

Reviewer #1 (Remarks to the Author):

I found the article acceptable.

Reviewer #2 (Remarks to the Author):

The manuscript presents study of the influence of applied strain on micro-level magnetic properties for non-oriented electrical steels with magnetic force microscopy, transmission Kikuchi diffraction and 4D Scanning transmission electron microscopy. Magnetic structure behaviour was investigated within a single grain.

I find this manuscript well written and presented. The quality of data is good and it was obtained with appropriate experimental techniques and thoroughly analyzed. For example, authors used on-axis transmission Kikuchi diffraction geometry with horizontal screen that excludes the distortions (with increased spatial resolution in comparison to off-axis TKD, EBSD). TKD is a promising new technique for the analysis and routine characterisation of nanocrystalline materials and nanostructures, capable of effective resolutions of down to few nanometres.

Novelty, motivation of the research and provided results are clear and well explained. Especially, I find Figure 2 very helpful for the reader because it makes clear what information was obtained with different experimental techniques and explains the structure of experimental part of the manuscript.

The authors convinced me that those results have potential significance for the field of manufacturing of the electrical machines because, procedures that are applied during production can influence the magnetic domains at the microstructural level and should be taken into account.

I do not have any doubts about the validity of the approaches, that were used for investigation, and the quality of the obtained data, but I would like to ask the authors to provide more clear evidence of their claims. Namely:

-In the text before Fig. 5 authors state “A possible explanation for the load jump (for HSR) is that strain was transferred deeper into the bulk material, inducing plastic deformation before dislocations were able to accumulate in the region directly under the primary deformation zone (Figure 5f-iii). This indicates that the region with a low misorientation angle (Figure 5d, Figure 5f-iv) is only deformed elastically when transmitting the strain to the bulk material.“

It would be nice if authors explained this statement in more details: it is unclear to me why in case of HSR strain was transferred deeper including plastic deformations but on the other hand the region with small misorientation angle is ONLY deformed elastically. It is even more confusing later when authors claim ”The contour beneath the primary zone which is surrounded by plastic deformation (Figure 5f-iv), shows a relative increase in magnetisation, with a demagnetisation band observed directly below, at the boundary (Figure 5f-v) with the secondary deformation zone (Figure 5f-vi)”. Because, in the Figure 5f-iv it is written that region iv is “Elastic transfer zone”.

-In the abstract authors write “… we reveal that low velocity indentations result in magnetic deflections away from the deformed zone when compared with the higher velocity which has a higher degree of alignment due to defect-pinning“. But if one compares Figure 5 (g and h) for LSR and HSR respectively, it could be seen that magnetic deflection appears at the same coordinates for Z and X. They maybe oriented parallel to X in case of HSR and parallel to Z in case of LSR but, still one can find red regions (that correspond to high β_L) across the whole acquisition area. Furthermore, in Figure 5 (h) there are no areas with high β_L close to indent surface in contrary to Figure 5 (g) that corresponds to LSR. Please, clarify this in more details.

I have some suggestions that from my point of view could improve the manuscript:

-Please, state the spatial resolution for TKD measurements. I assume, that with the thickness of the lamellae about 150nm, accelerating voltage of 30kV and electron beam current of 1.0 nA, spatial resolution is about 20nm or higher?

-It would be interesting to see Kikuchi patterns for the selected single grain for LSR and HSR at least in the Supplementary materials. Are there any differences between them?

-Does colour-code for Figure 5 (c-d) correspond to the colour-code of the stereographic triangle in Figure 2? Please clarify this in the caption for Figure 5.

-Maybe one can add to supplementary material a plot that represents statistics of the EBSD-determined grain sizes?

-Please, check one more time notations in the text (numbers and letters for figures). There are my suggestions:

Line 240: the abbreviation “TKD GROD” appears in the capture of Figure 5 earlier than it is explained in the text;

Line 244: colour bar for (g) and (h)?

Line 251: For HSR (Figure 5 h)?

Line 265: Tying the cross-sectional (||XZ) observations presented in Figure 5 ?

Line 266: free-surface (||XY), shown in Figures 3 and 4?

Reviewer #3 (Remarks to the Author):

The manuscript reports on a study on the effect of mechanical deformations on microscale magnetic behavior of non-oriented electrical steel. The main strength of the work is undoubtedly represented by the synergistic approach which takes advantage of the use of different techniques allowing the selection of single grains, their indentation, the magnetic mapping, and the cross sectional analysis under the indented area.

Despite this interesting approach, the manuscript does not contains a clear description of the results and a comprehensive rationalization of the results. Also, although the results can be interesting, they do not seem to justify their publication in Nature Communications. I therefore would recommend the rejection of the manuscript, although a resubmission can be considered after a careful revision of the manuscript. Below my comments as I found them reading the manuscript.

- overall, the English writing a significant improvement. Also the structure of some sentences seems broken and should be revised.

- the introduction starts with a figure which is decorative but without a practical importance for the manuscript, followed by a sort of graphical abstract describing the scope of the work. This is definitely confusing and should be rationalized.

- the introduction end with the sentence “Not to be regarded as a scope of the study but…” which adds a further element of confusion. It would be better deleted or at least moved in the discussion.

- the “Results” section is actually a not so rational merge of experimental section, with materials and methods, and results. The structure should be rationalized, for instance reporting what is actually a “materials and methods” topic in the next session.

- the authors asses they selected the <111> direction grain. How was this done? Do they identified the grain “outside” the nanoindenter and then moved the sample or was it “in situ”? Please detail. I assume the procedure is the one described later about MFM, but please clarify.

- line 130: “The details of the investigatory procedure are detailed in Figure 2 and the methods.” please fix this sentence.

- line 144: “before the MFM phase distribution shows a gradient between opposing polarities”: what does it means? Are the authors referring to a smooth variation? Maybe some profiles could help.

- together with MFM images, topography images of the surface should be added. In particular because the topography may often induce electrostatic artifacts in MFM phase images and thus the possible morphology contribution to the MFM map should be discussed.

- The authors report the roughness measured as to be 0.447 ± 0.0725 nm. It is not clear the importance of this parameter in the work. Also, if the information is maintained, please rationalize the significant digits of the mean and the uncertainty.

- the method for the analysis of the radial extent of the magnetic is interesting. However, some clarifications are needed, e.g., “The indent footprint itself was also removed from the annular subsets to avoid the inclusion of MFM…” what does it mean?

- the method requires the integration over an circular region. Is the anisotropy relevant for this method? I mean, a large scale anisotropy due to the different grain orientation…

- it is not clear which phase shift the authors use: is it the absolute shift or is the phase after subtraction of the mean (as it seems from Fig. 3)?

- in supplementary materials, please note that MFM images are referred to as AFM.

Reviewer #4 (Remarks to the Author):

This manuscript aims to demonstrate that deformation resulted from indentation at different stain rates affects the magnetic domain distribution. Characterization techniques such as MFM, TKD and STEM/DPC were used. The re-arrangement of magnetic domains in a small area under stress/strain application has long been demonstrated (for example, Zhang et al., Nuclear Instruments and Methods in Physics Research B, 2007, 257, 379-382). This study,

however, failed to further correlate plastic strain and domain re-arrangement. For example, high GROD values for the HSR sample are observed near the surface as compared to those for the LSR one, whereas higher MFM phase shifts are found for the latter. Moreover, the high GROD values mentioned above should result in a higher rotation of the magnetic moments. The projection of the saturated magnetization (Bs) on the sample surface and resulted change of magnetic deflection angle (beta L) should vary from one position to another on surface. However, the DPC map does not correspond to the argument. Accordingly, this manuscript shows preliminary attempts to analyze local changes of magnetic domain structure on strain, but more efforts are needed to quantify the results.

Response to Reviewer #1:

General comments:

I found the article acceptable.

Response to general comments:

Thank you for taking the time to review our manuscript.

Specific comments:

N/A

Response to Reviewer #2 (Amendments are marked in cyan):

General comments:

The manuscript presents study of the influence of applied strain on micro-level magnetic properties for non-oriented electrical steels with magnetic force microscopy, transmission Kikuchi diffraction and 4D Scanning transmission electron microscopy. Magnetic structure behaviour was investigated within a single grain.

I find this manuscript well written and presented. The quality of data is good and it was obtained with appropriate experimental techniques and thoroughly analyzed. For example, authors used on-axis transmission Kikuchi diffraction geometry with horizontal screen that excludes the distortions (with increased spatial resolution in comparison to off-axis TKD, EBSD). TKD is a promising new technique for the analysis and routine characterisation of nanocrystalline materials and nanostructures, capable of effective resolutions of down to few nanometres.

Novelty, motivation of the research and provided results are clear and well explained. Especially, I find Figure 2 very helpful for the reader because it makes clear what information was obtained with different experimental techniques and explains the structure of experimental part of the manuscript.

The authors convinced me that those results have potential significance for the field of manufacturing of the electrical machines because, procedures that are applied during production can influence the magnetic domains at the microstructural level and should be taken into account.

Response to general comments:

Thank you for taking the time to review our manuscript and for providing constructive comments. We would like to clarify that the TKD was achieved using off-axis geometry. While the authors agree that using on-axis TKD geometry would yield advantages regarding EBSP distortion, due to equipment constraints, we used off-axis geometry. Although this technique introduces EBSP distortion, accurate indexing can still be achieved.

With respect to (the previously named) Figure 2 in the original manuscript, thank you for your favourable comments. To support comments made by Reviewer #3, we have rationalised (the previously named) Figure 1 by merging it with Figure 2 which forms a new Figure 1. We have kept all the elements of the previous Fig. 2 in the new merged figure, so that the message to the reader is clear.

Amendments in the revised manuscript on page 18 – Response to Reviewer #2 general comments:

To map the sub-granular deformation occurring beneath the indentations and compressed pillars, off-axis TKD was performed with an accelerating voltage of 30 kV, an electron beam current of 1.0 nA, and a pixel size of 0.02 μm.

Amendments in the revised manuscript on page 5 – Response to Reviewer #2 general comments:

New Figure 1 (By merging Figure 1 and Figure 2):

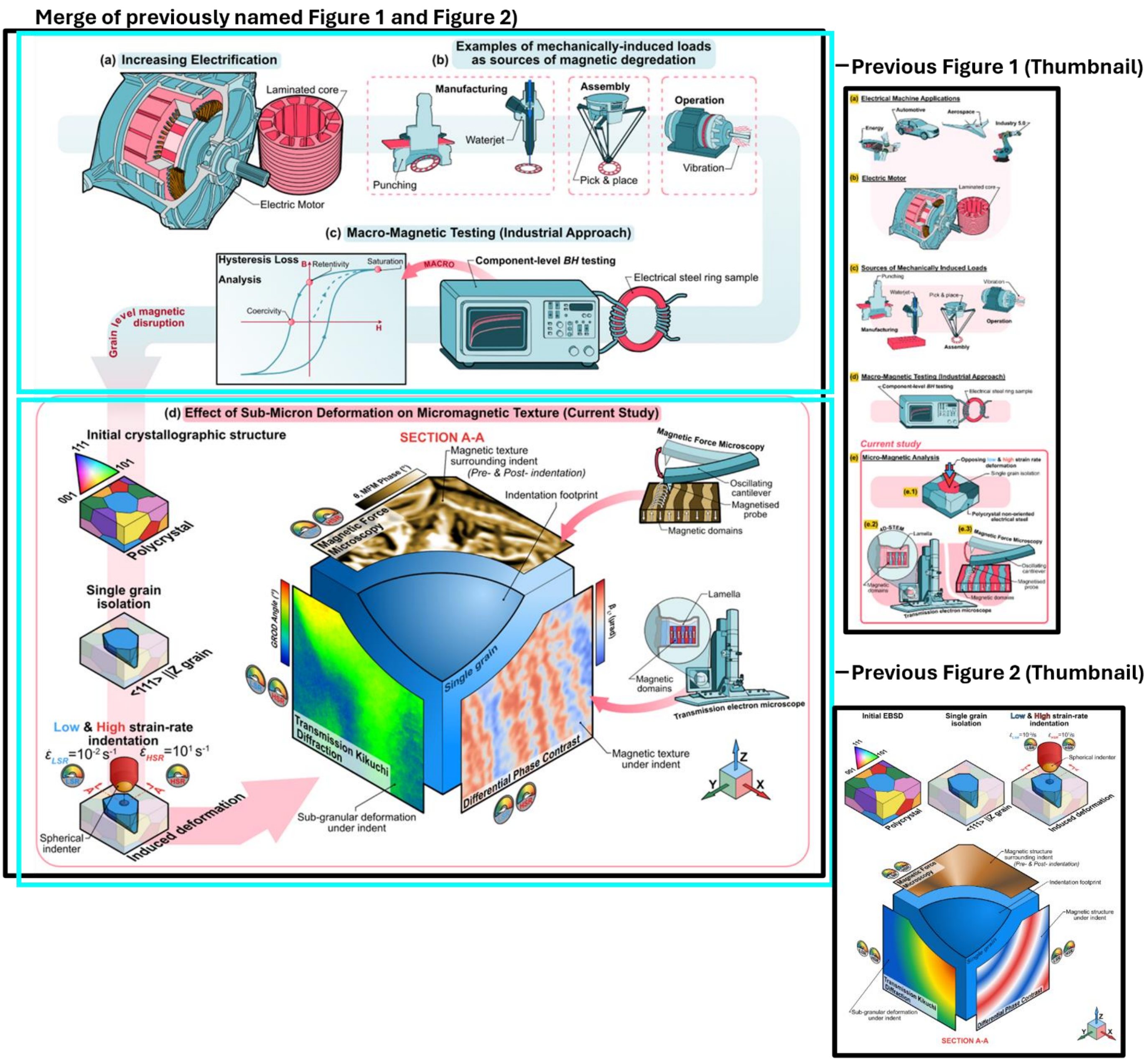

Figure 1 **Scope of the investigation. a** Electric motor application. **b** Example sources of mechanically induced loads experienced by electrical steel laminations from manufacture to assembly, and its operational life. **c** Conventional macro-level *BH* magnetic testing completed at the component level which is commonly used for measuring the magnetic performance of electrical steel laminations. **d** Schematic overview of the experimental procedure. Showing the selection of a single grain through EBSD inverse pole figure mapping (with associated stereographic key) and the induced deformation at low (LSR) and high (HSR) strain rates. A schematic representation of the data-cube is shown with magnetic force microscopy on the free-surface (||XY) and transmission Kikuchi diffraction and differential phase contrast being used to investigate the material deformation and magnetic domain structure respectively.

Specific Comments:

In the following we have grouped 3 comments from Reviewer 2 as they refer to elements which have been superseded by the content in the revised manuscript.

Comment R2-1:

I do not have any doubts about the validity of the approaches, that were used for investigation, and the quality of the obtained data, but I would like to ask the authors to provide more clear evidence of their claims. Namely:

In the text before Fig. 5 authors state “A possible explanation for the load jump (for HSR) is that strain was transferred deeper into the bulk material, inducing plastic deformation before dislocations were able to accumulate in the region directly under the primary deformation zone (Figure 5f-iii). This indicates that the region with a low misorientation angle (Figure 5d, Figure 5f-iv) is only deformed elastically when transmitting the strain to the bulk material.“

It would be nice if authors explained this statement in more details: it is unclear to me why in case of HSR strain was transferred deeper including plastic deformations but on the other hand the region with small misorientation angle is ONLY deformed elastically.

Comment R2-2:

It is even more confusing later when authors claim ”The contour beneath the primary zone which is surrounded by plastic deformation (Figure 5f-iv), shows a relative increase in magnetisation, with a demagnetisation band observed directly below, at the boundary (Figure 5f-v) with the secondary deformation zone (Figure 5f-vi)”. Because, in the Figure 5f-iv it is written that region iv is “Elastic transfer zone”.

Comment R2-3:

Furthermore, in Figure 5 (h) there are no areas with high β_L close to indent surface in contrary to Figure 5 (g) that corresponds to LSR. Please, clarify this in more details.

Consolidated response to comments R2-1:3:

Thank you for the constructive critique. Comments R2-1:3 refer to the results and subsequent discussion presented in the cross-sectional observations in what was previously Figure 5 (now Figure 3). We have provided a consolidated answer based on a closer observation of the results and through the inclusion of further experimental evidence in the form of micro-pillar compression, followed by cross-sectional TKD and 4DSTEM experiments (Figure 4 in the revised manuscript).

In the text referred to in comment R2-1, we originally attempted to explain the horizontal band in the DPC image in the initial manuscript through work hardening, causing the deformed bulk material directly under the indentation to act as a secondary indenter, thus, deforming the material deeper below the free-surface. In this case, we posited that the horizontal band of low GROD misorientation transferred the kinetic energy of the bulk deeper while not having enough time to accommodate the dislocations at HSR. We agree with comment R2-2 that the explanation becomes more confusing when we refer to the different regions as arbitrary zones in the previously named Figure 5, insets e, f (below):

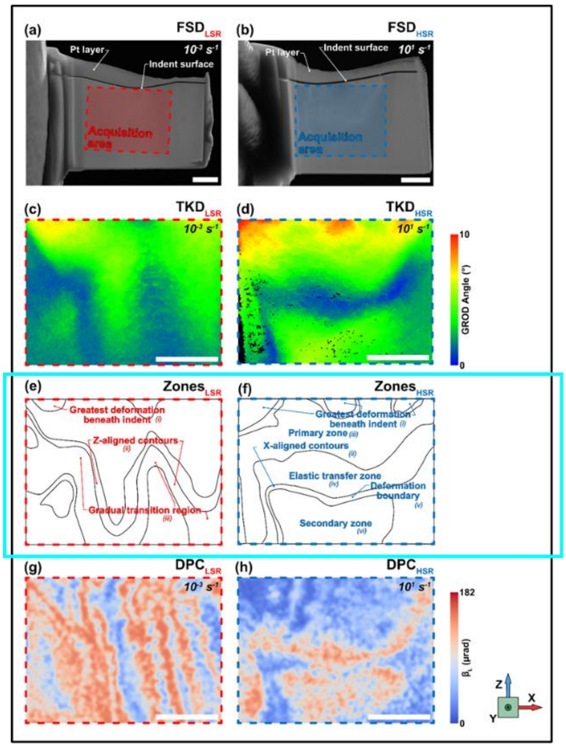

Previous Figure 1 (Thumbnail)

Therefore, in the revised manuscript, we have removed the previous diagrams e, f (above) in the revised Figure 3 and have re-written the discussion to focus on slip system direction as opposed to the pure deformation ‘magnitude’ as explained through TKD GROD angle analysis.

Additionally, we have completed further experiments to provide more evidence which have enabled us to better explain our initial observations. We generated multiple micro-pillars within a single grain and compressed these similarly at low and high strain rates. Besides micro-pillars allowing for deformation which is not constrained by the bulk material (as in spherical nano-indentations), and applying the load with a constant projected area, we opted to load the crystal structure in a different direction (parallel to direction <100> as opposed to <111>) to investigate how orientation and subsequent slip system activation could influence the magnetic texture. These results are shown in the newly added Figure 4 in the manuscript.

Amendments in the revised manuscript on page 10 – Response to comments R2-1:3:

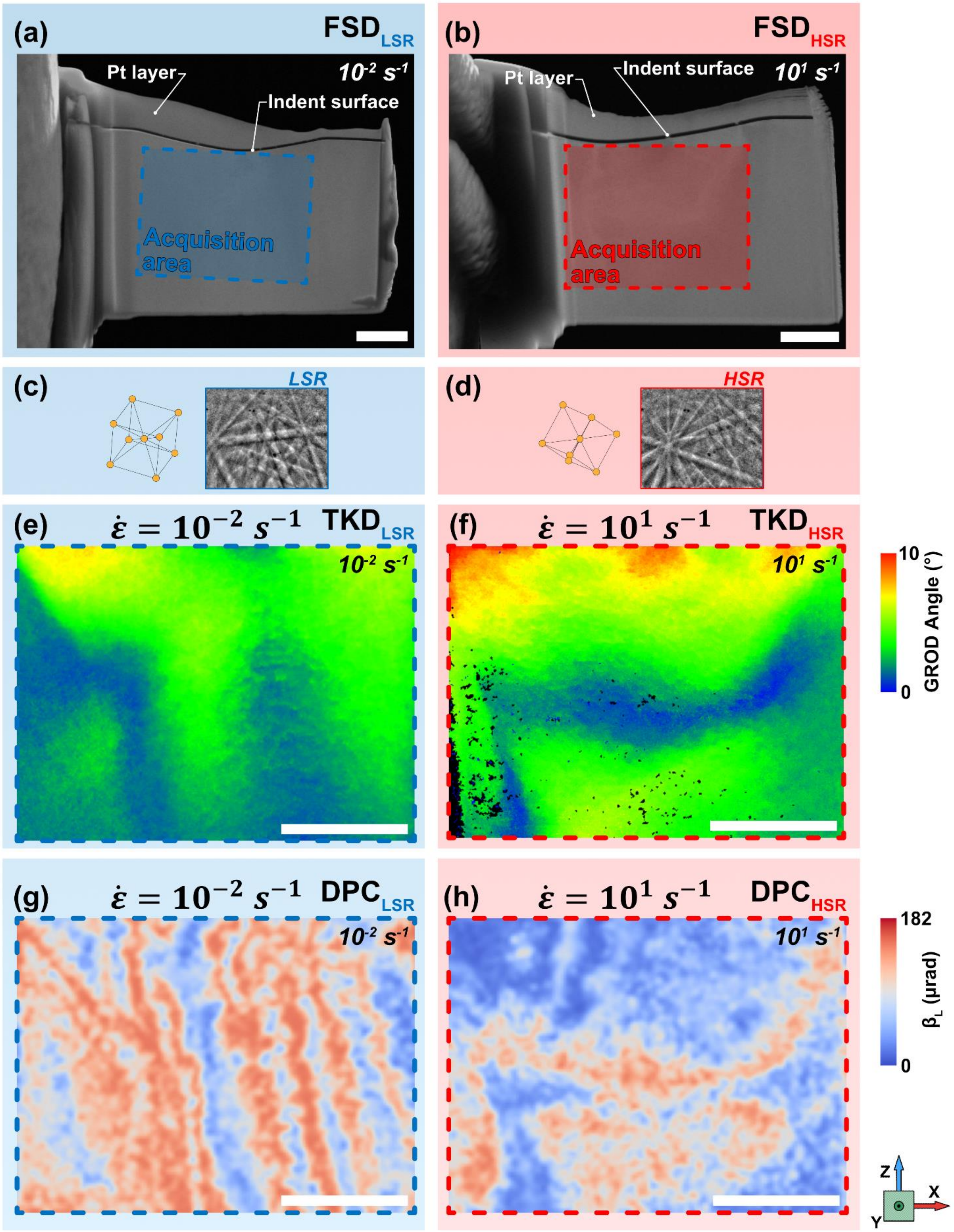

Figure 3 The effect of mechanically induced deformation on in-plane magnetic deflection within single grains. **a, b** FSD images of the extracted lamellae, cross-sectioning the indentations, made at low (0.01 s^{-1}) and high (10.00 s^{-1}) strain rates respectively. **c,d** Extracted Kikuchi patterns and corresponding Fe-BCC unit cell representation from the cross-sectioned regions. **e, f** TKD GROD angle maps revealing sub-granular deformation by means of crystallographic misorientation. Map locations are the same as the acquisition areas annotated in **a, b**, respectively. Colour bar for both **e, f** indicates the GROD angle at the same scale. **g, h** STEM DPC images showing the in-plane (||XZ) magnetic deflection in the same deformation regions as in **e, f**, respectively, and indication of strain-rate dependence of the magnetic domain structure. Colour bar for both **g, h** gives the magnitude of in-plane (||XZ) magnetic deflection angle, β_L, at the same scale. The coordinate system shown in **a** is the same for all subfigures **a,b,e,f,g,h**. Scale bars in subfigures **a,b,e,f,g,h**, 2 μm.

Amendments in the revised manuscript on page 11-12 – Response to comments R2-1:3:

To understand the effects that mechanically induced deformation at low and high strain-rates has on the magnetic texture, we performed scanning transmission electron microscopy – differential phase contrast (STEM-DPC) (Figure 3g, h) with a pixellated detector to map the magnitude of the in-plane magnetic deflection, β_L, beneath the indentations (see more details in the methods section).

In LSR indentation, plastic deformation is focussed on a lower number of primary slip systems, which require a lower energy threshold for activation compared to the secondary slip systems [28]. As the grain was loaded in the <111> direction, the {111} family of slip planes (the primary slip planes in BCC crystals) were easily activated. The more vertically oriented bands observed in the DPC map (Figure 3g) suggests a correlation between the direction of the deformation network and the arrangement of magnetic structures.

Conversely, in HSR indentation, the activation energy conditions for multiple, secondary slip systems may be activated during dynamic loading. The activation of these secondary slip systems causes diffusion of the plastic deformation, which leads to directionally shifted magnetic structures observed in the two-dimensional cross-section view (Figure 3h). As the dynamic HSR indentation with a greater input energy can activate the secondary slip systems deeper below the indent surface, horizontal contours in the TKD map (Figure 3f) have been observed. Despite the region immediately below the indentation surface having a greater magnitude of deformation than that which occurred deeper, the corresponding DPC map (Figure 3h) highlights that there is a stronger relationship between the direction of the activated slip systems and the magnetic alignment than with the deformation magnitude. This activation of non-primary slip systems in the HSR sample, coupled with the simultaneous occurrence of both shearing and compression during fast deformation events gives rise to a greater number of boundaries at which the magnetic structures can accumulate.

Tying information on the magnetic domains in the cross-sectional (||XZ) observations presented in Figure 3 with those at the free-surface (||XY), shown in Figure 2, we can conclude that the strain-rate of applied deformation has a significant, time-dependent effect on the ability of the material to accommodate stresses. This has a marked effect on the magnetic textures at the level of a single grain. Applications of LSR loads were found to induce deformation which primarily altered the magnetic domain texture at the free-surface (||XY) – Figure 2i (see *Post-LSR* line). This may be attributed to the orientation of the activated primary slip system intersecting with the surface, allowing the domain pinning effects to be observed on the external surface. However, for HSR load application, occurring with an increase in indentation energy, non-preferentially aligned slip systems can be activated. In this case, the deformation boundaries which magnetic structures were observed to align with are situated deeper below the free-surface (||XY) (shown in the cross-section – Figure 3h). This had the effect of an apparent reduction in $M\theta_A$ on the free-surface (||XY), requiring cross-sectional observation to determine the arrangement of the magnetic structures – Figure 2i (see *Post-HSR* line).

Amendments in the revised manuscript on page 13 – Response to comments R2-1:3:

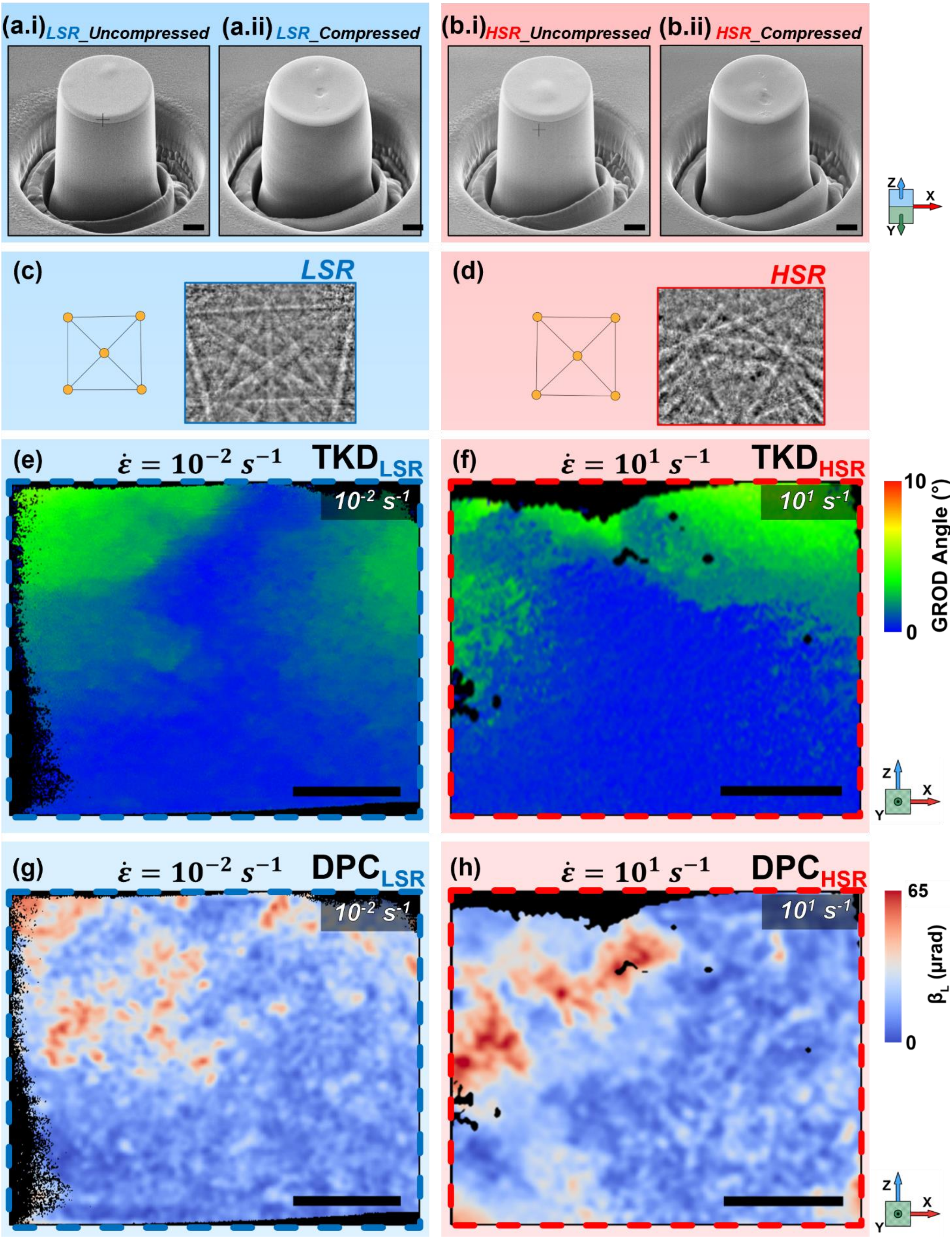

Figure 1 The effect of mechanically induced deformation on in-plane magnetic deflection within compressed pillars located in a single grain. a, b SEM images showing the pillars which were compressed at low (0.01 s^{-1}) and at comparatively higher strain rates (10.00 s^{-1}) before (**a.i, b.i**) and after (**a.ii, b.ii**) compression, respectively. **c, d** Extracted Kikuchi patterns and corresponding Fe-BCC unit cell representation from the cross-sectioned regions. **e, f** TKD GROD angle maps revealing sub-granular deformation by means of crystallographic misorientation. The lamellae for **e, f** were extracted by cross-sectioning (in the ||XZ plane) the compressed pillars shown in **a.ii, b.ii**, respectively. Colour bar for both **e, f** indicates the GROD angle at the same scale. **g, h** STEM DPC images showing the in-plane (||XZ) magnetic deflection in the same compressed regions as in **e, f**, respectively. Colour bar for both **g, h** gives the magnitude of in-plane (||XZ) magnetic deflection angle, β_L, at the same scale. The coordinate systems for the subfigures are given by the triad on the right of each row. Scale bars in all subfigures **a,b,e,f,g,h**, 1 µm.

Amendments in the revised manuscript on page 12-14 – Response to comments R2-1:3:

To provide more evidence for the observations discussed around the changes of magnetic domains within plastically deformed regions, we have performed further tests in different loading conditions. To simplify the loading scenario, and to avoid a complex stress distribution akin to that which occurred in the conventional spherical indentation (as above), we compressed a set of micro-pillars (Figure 4a.i, b.i – pre-compression, Figure 4a.ii, b.ii – post-compression at LSR and HSR respectively) which were located within a single grain oriented with the <100> direction of the crystal parallel to the defined X, Y, and Z axes. Compressing pillars ensures that the loaded area of the grain, hence the magnetic texture, is isolated from the neighbouring material. The pillars were deformed plastically to the same strain ($\varepsilon = 0.078$) at opposing strain rates. Like the spherical indents, we compressed the pillars plastically at both LSR (10^{-2}) and HSR (10^{1}) to isolate and provide more evidence to how the different deformation mechanisms influence the distribution of magnetic textures. To further support the discussions referring to slip system activation when referring to the changes in magnetic textures, we performed LSR and HSR tests on the pillars at a different crystal orientation (<100>) from that used in the previously discussed spherical indentations (<111>). As such, we would not expect the deformation observed from TKD nor the magnetic alignment to occur in the same directions.

For both LSR (Figure 4e) and HSR (Figure 4f) pillar compression, the deformation from the TKD maps predominantly aligns with the primary slip planes in the <111> direction. The corresponding DPC map for LSR pillar compression (Figure 4g) shows a weak banded magnetic structure following this alignment indicating that, like in the quasi-static indentations (LSR), the plastic deformation can accumulate along the easily activated primary slip planes. However, as the pillar compression energy increases at HSR, secondary slip systems can be activated, inducing alignment in different directions, resulting in a more-complex deformation network (Figure 4f). This activation of secondary slip systems at HSR (Figure 4h) presents more boundary options for the magnetic structures to align with.

Comment R2-4:

I have some suggestions that from my point of view could improve the manuscript:

Please, state the spatial resolution for TKD measurements. I assume, that with the thickness of the lamellae about 150nm, accelerating voltage of 30kV and electron beam current of 1.0 nA, spatial resolution is about 20nm or higher?

Response to comment R2-4

Thank you for your comment, the pixel size used for the LSR TKD was 20nm, and for HSR TKD was 14.6nm. As the grain-referenced method was used for measuring misorientation instead of a local/kernel method, these results can be compared. We have included the pixel size in the methods section as the greater value: 0.02 µm.

Amendments in the revised manuscript on page 18 – Response to comments R2-4:

To map the sub-granular deformation occurring beneath the indentations and compressed pillars, off-axis TKD was performed with an accelerating voltage of 30 kV, an electron beam current of 1.0 nA, and a pixel size of 0.02 µm.

Comment R2-5:

It would be interesting to see Kikuchi patterns for the selected single grain for LSR and HSR at least in the Supplementary materials. Are there any differences between them?

Response to comment R2-5

Thank you for your comment. We have included extracted Kikuchi patterns from the TKD lamellae for LSR and HSR indentation (Figure 3 c, d) as well as the TKD for LSR and HSR micro-pillar compression (Figure 4 c, d).

Amendments in the revised manuscript on page 10 – Response to comments R2-5:

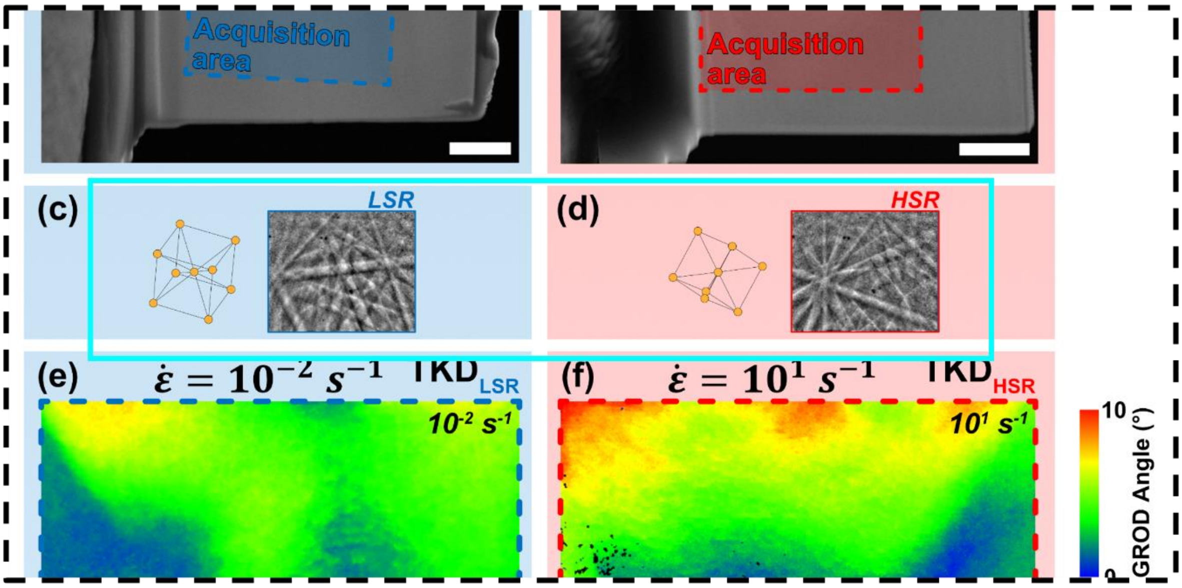

Amendments in the revised manuscript on page 14 – Response to comments R2-5:

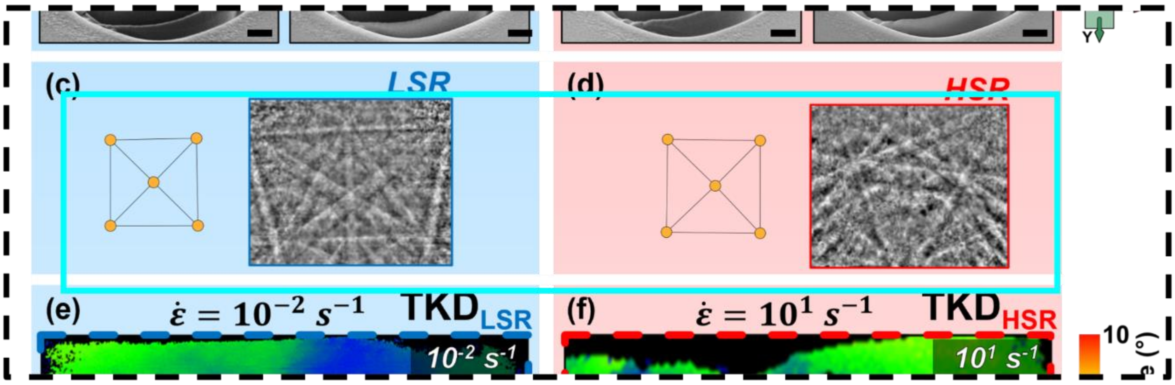

Comment R2-6:

Does colour-code for Figure 5 (c-d) correspond to the colour-code of the stereographic triangle in Figure 2? Please clarify this in the caption for Figure 5.

Response to comment R2-6

Thank you for comment. The colour code for the previously named Figure 5c-d did not correspond to the stereographic triangle in the previously named Figure 2 (inserted below for reference).

Previously in Figure 2

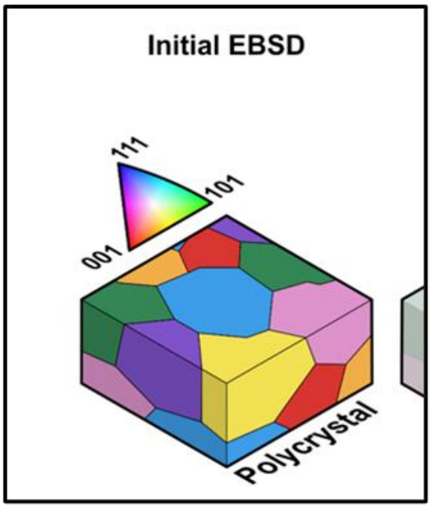

Previously in Figure 5

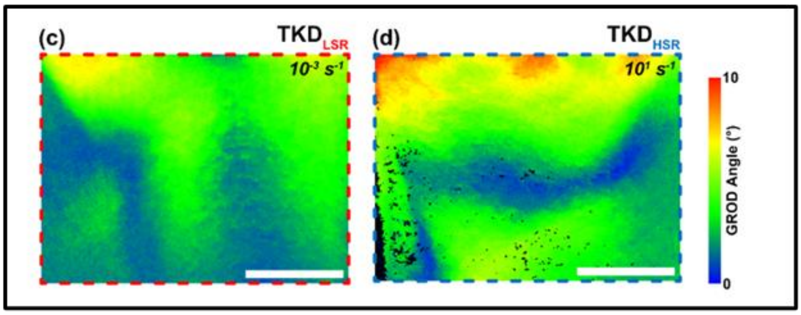

The stereographic triangle related to the inverse pole figure map whereas the colour bar related to the value of GROD angle. We have added clarification for this in the caption for Figure 1.

Amendments in the revised manuscript on page 5 – Response to comments R2-6:

Figure 2 Scope of the investigation. a Electric motor application. b Example sources of mechanically induced loads experienced by electrical steel laminations from manufacture to assembly, and its operational life. c Macro-level *BH* magnetic testing completed at the component level which is commonly used for measuring the magnetic performance of electrical steel laminations. d Schematic overview of the experimental procedure. Showing the selection of a single grain through EBSD inverse pole figure mapping (with associated stereographic key) and the induced deformation at low (LSR) and high (HSR) strain rates. A schematic representation of the data-cube is shown with magnetic force microscopy on the free-surface (||XY) and transmission Kikuchi diffraction and differential phase contrast being used to investigate the material deformation and magnetic domain structure respectively.

Comment R2-7:

Maybe one can add to supplementary material a plot that represents statistics of the EBSD-determined grain sizes?

Response to comment R2-7

Thank you for comment. We have included a grain-size statistical plot in the supplementary materials, S4-b.i. We have shown this below, with the specific subfigure highlighted in cyan.

Amendments in the revised manuscript in the supplementary materials, S4 – Response to comments R2-7:

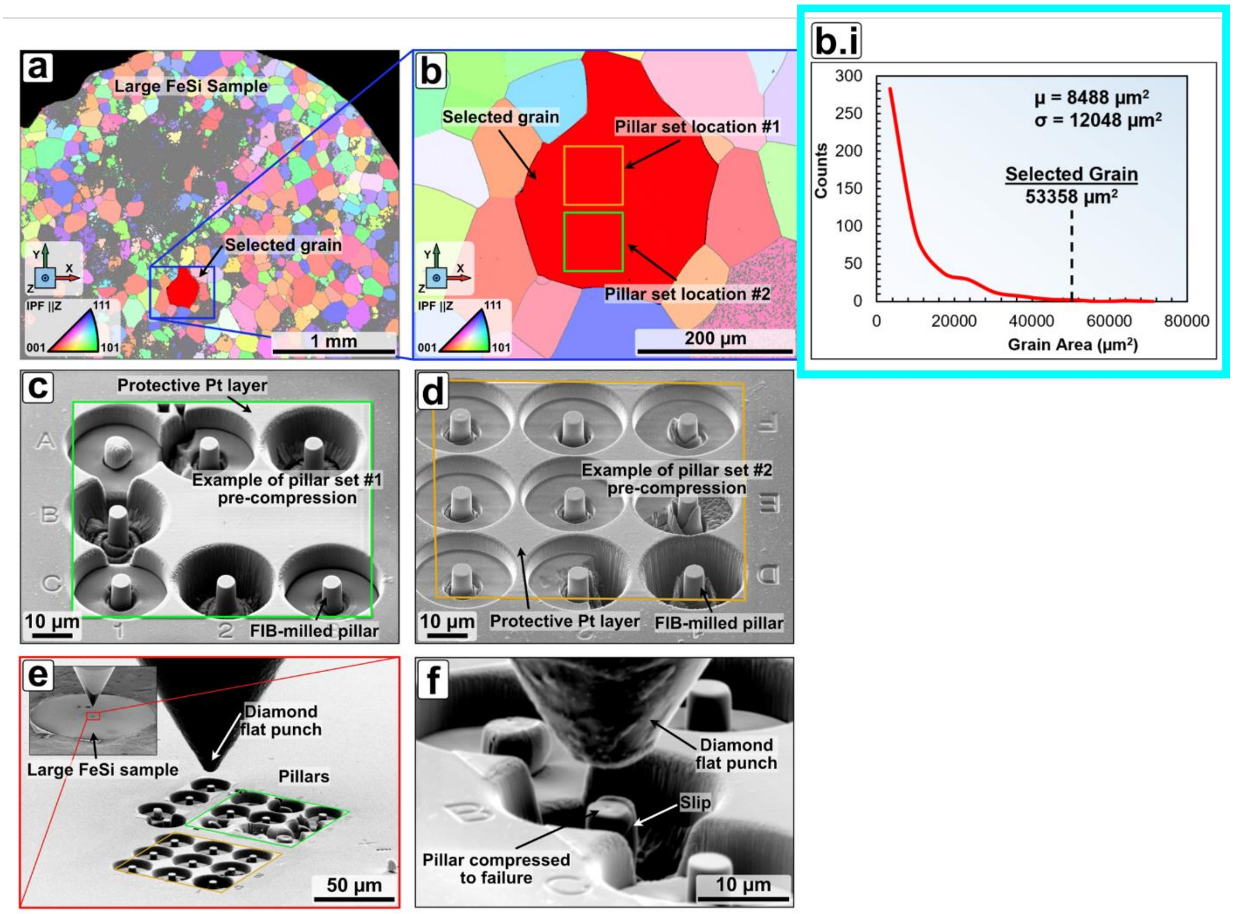

Figure 10 Expanded micro-pillar procedure: Locating and FIB milling. a Preliminary, coarse EBSD inverse pole figure map used to identify potential grain candidates. b Increased resolution EBSD inverse pole figure map with the selected grain highlighted. b.i, Grain size statistical plot with the area of the selected grain from b marked with a dotted line. c, d Regions where micro-pillars were manufactured within green and orange squares located in the single grain shown in b, respectively. e Overview in-situ SEM image showing the diamond flat punch positioned over the micro-pillars pre-compression. f SEM image taken post-compression of a micro-pillar which has been compressed to failure showing slip.

Amendments in the revised manuscript on page 16 – response to comment R2-7:

A large grain (area = 53,358 μm^2 shown in Figure S4-b.i, in the supplementary materials) was identified for pillar manufacture (Figure 5b) with orientation (<100> ||Z) so that it can accommodate many pillars while avoiding the errors that might be introduced by grain boundaries.

Comment R2-8:

Please, check one more time notations in the text (numbers and letters for figures). There are my suggestions:

Comment R2-9:

Line 240: the abbreviation “TKD GROD” appears in the capture of Figure 5 earlier than it is explained in the text;

Response to comment R2-9

Thank you, the inclusion of the “GROD” abbreviation has now been included when TKD appears in the text page 9.

Amendments in the revised manuscript on page 9 – Response to comments R2-9:

Through transmission Kikuchi diffraction (TKD) grain-referenced orientation deviation (GROD) mapping, …

Comment R2-10:

Line 244: colour bar for (g) and (h)?

Response to comment R2-10

Thank you. The previously named Figure 5 has become Figure 3 and the figure caption has been corrected on page 10-11.

Amendments in the revised manuscript on page 10-11 – Response to comments R2-10:

Colour bar for both g, h gives the magnitude of in-plane (||XZ) magnetic deflection angle, β_L, at the same scale.

Comment R2-11:

Line 251: For HSR (Figure 5 h)?

Response to comment R2-11

Thank you. This section has been replaced in text with the discussion referenced in response to comments R2-1:3.

Comment R2-12:

Line 265: Tying the cross-sectional (||XZ) observations presented in Figure 5 ?

Comment R2-13:

Line 266: free-surface (||XY), shown in Figures 3 and 4?

Response to comment R2-12,13

Thank you. This section has been modified in text with the discussion referenced in response to comments R2-1:3 and the cross-reference to the correct figure has been checked for accuracy.

Amendments in the revised manuscript on page 11 – Response to comments R2-12,13:

Tying information on the magnetic domains in the cross-sectional (||XZ) observations presented in Figure 3 with those at the free-surface (||XY), shown in Figure 2, we can conclude…

Response to Reviewer #3 (Amendments are marked in green):

General Comments

The manuscript reports on a study on the effect of mechanical deformations on microscale magnetic behavior of non-oriented electrical steel. The main strength of the work is undoubtedly represented by the synergistic approach which takes advantage of the use of different techniques allowing the selection of single grains, their indentation, the magnetic mapping, and the cross sectional analysis under the indented area.

Despite this interesting approach, the manuscript does not contains a clear description of the results and a comprehensive rationalization of the results. Also, although the results can be interesting, they do not seem to justify their publication in Nature Communications. I therefore would recommend the rejection of the manuscript, although a resubmission can be considered after a careful revision of the manuscript.

Response to general comments:

Thank you for reviewing our manuscript and providing helpful feedback. Regarding the comment on the original manuscript not containing a clear description of the results nor comprehensive rationalisation of the results, we have conducted further experiments which have enables us to provide a clearer discussion.

We have conducted a significant amount of further experimentation in new conditions which have enabled us to refine the results observed. By employing micro-pillar compression, in a grain with a different orientation to the initial trials (loading parallel to <100> vs <111>, respectively) we have been able to tie our observations with discussions on slip systems activated in the Fe-BCC structure. Specifically, we have progressed the discussion away from a commentary on the magnitude of plastic deformation and it's correlation with the magnetic texture. In the revised manuscript, we have focussed the discussion on the activation of primary and secondary slip systems and how the magnetic texture responds to the direction of the plastic deformation. We also address this in the earlier response to comments R2-1:3.

Specific Comments:

Comment R3-1:

overall, the English writing a significant improvement. Also the structure of some sentences seems broken and should be revised.

Response to comment R3-1:

Thank you for the comment. The revised manuscript has been proof-read again by peers and sentence structure/phrasing has been simplified throughout to improve readability.

Comment R3-2:

the introduction starts with a figure which is decorative but without a practical importance for the manuscript, followed by a sort of graphical abstract describing the scope of the work. This is definitely confusing and should be rationalized.

Response to comment R3-2:

Thank you for the constructive critique. The intention of the previously named Figure 1 was to help convey the wider research problem and it's impact to the reader. The previously named Figure 2 was included with the intention of conveying our synergistic approach to the reader in a self-explanatory manner. However, we agree that their inclusion should be rationalised. Therefore, we have merged the previously named Figures 1 and 2 and rationalised them as Figure 1 in the revised manuscript.

Amendments in the revised manuscript on page 5 – Response to comments R3-2:

New Figure 1 (By merging Figure 1 and Figure 2):

Merge of previously named Figure 1 and Figure 2)

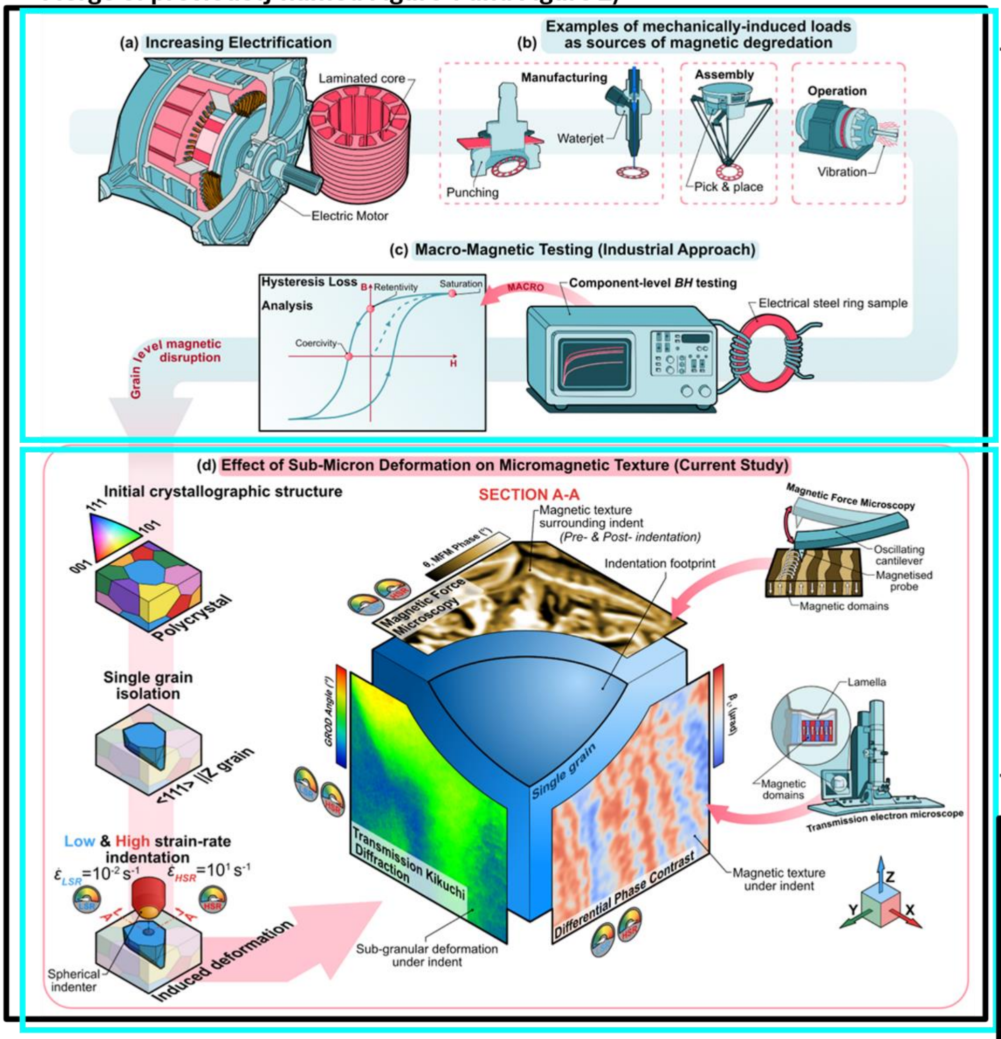

Previous Figure 1 (Thumbnail)

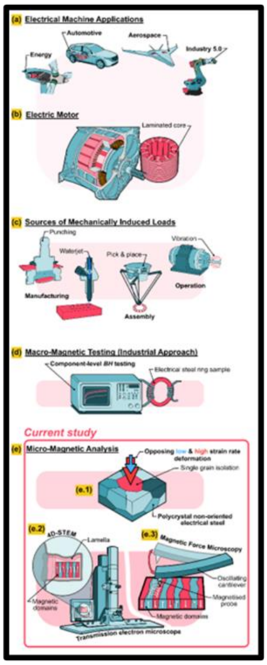

Previous Figure 2 (Thumbnail)

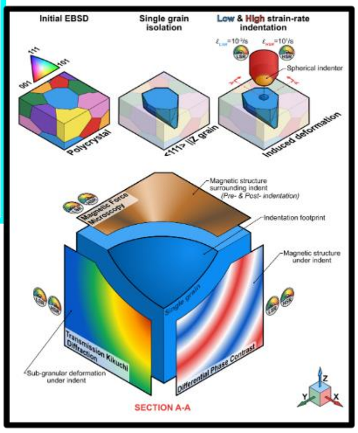

Figure 1 **Scope of the investigation.** a Electric motor application. b Example sources of mechanically induced loads experienced by electrical steel laminations from manufacture to assembly, and its operational life. c Macro-level BH magnetic testing completed at the component level which is commonly used for measuring the magnetic performance of electrical steel laminations. d Schematic overview of the experimental procedure. Showing the selection of a single grain and the induced deformation at low (LSR) and high (HSR) strain rates. A schematic representation of the data-cube is shown with magnetic force microscopy on the free-surface (||XY) and transmission Kikuchi diffraction and differential phase contrast being used to investigate the material deformation and magnetic domain structure respectively.

Comment R3-3:

the introduction end with the sentence "Not to be regarded as a scope of the study but…" which adds a further element of confusion. It would be better deleted or at least moved in the discussion.

Response to comment R3-3:

Thank you for the comment. The text has been amended in the revised manuscript on page 3 to avoid confusion.

Amendments in the revised manuscript on page 3 – Response to comments R3-3:

The study of the relationship between the application of micro-loads and the corresponding magnetic domain disturbances could reveal the governing mechanisms which lead to increased electrical losses in NOES (Figure 1d).

Comment R3-4:

the "Results" section is actually a not so rational merge of experimental section, with materials and methods, and results. The structure should be rationalized, for instance reporting what is actually a "materials and methods" topic in the next session.

Response to comment R3-4:

Thank you for the comments. The results section has been renamed: 'Results and discussion' on page 4. The intention of including some of the rationale behind the experimental methodology alongside the results was to communicate the required information to the reader at an appropriate point in the discussion.

Amendments in the revised manuscript on page 4:

Results and discussion

Comment R3-5:

the authors asses they selected the <111> direction grain. How was this done? Do they identified the grain "outside" the nanoindenter and then moved the sample or was it "in situ"? Please detail. I assume the procedure is the one described later about MFM, but please clarify.

Response to comment R3-5:

Thank you for the comment. The *<111>* oriented grain was selected using EBSD in a FEG-SEM prior to loading the sample into the nano-indenter located in a different, large chamber ESEM. We have clarified this in-text on page 4 with additional information in the Supplementary material (see amended Figure S1)

Amendments in the revised manuscript on page 4 – Response to comments R3-5:

Using a commercially available non-oriented electrical steel (Arnon 7 – FeSi 3 wt% Si), we induced mechanical deformation (see Figure 1d) in single grains with their normal axes along the <111> direction using a nano-indenter with a spherical diamond tip, allowing us to investigate the fundamental effects caused by a single loading scenario on the magnetic domain structure. The <111> oriented grain was selected from an initial EBSD map prior to loading the sample in the nano-indenter assembly.

Comment R3-6:

line 130: "The details of the investigatory procedure are detailed in Figure 2 and the methods." please fix this sentence.

Response to comment R3-6:

Thank you for the comment. The text has been amended on page 6.

Amendments in the revised manuscript on page 6:

The investigatory procedure is detailed in Figure 1d and the methods section.

Comment R3-7:

line 144: "before the MFM phase distribution shows a gradient between opposing polarities": what does it means? Are the authors referring to a smooth variation? Maybe some profiles could help.

<u>Response to comment R3-7:</u>

Thank you for the comment. By 'gradient between opposing polarities", the authors were referring to the rate of change between maxima and minima in the MFM phase maps. We have rephrased/simplified this statement to instead state how the MFM phase distribution becomes more polarised on page 6. We have also included line scans of the surface in the inset figures adjacent to the MFM maps in Figure 2 on page 7 (please see amendments made below).

Amendments in the revised manuscript on page 6 – Response to comments R3-7:

For both LSR and HSR indent locations, there is a visual difference in the magnetic domain structure before and after indentation – before, the MFM phase distribution shows less deviation from the mean in both the positive and negative directions (line scan insets of Figure 2a, d). After indentation, the MFM phase distribution becomes more polarised – deviating from the mean and having greater magnitudes of phase shift between extremum (line scan insets of Figure 2b, e) which indicates that there may be obstruction to magnetisation through domain motion/growth, favouring the more energetically demanding domain rotation [23].

Amendments in the revised manuscript on page 7 – Response to comments R3-7:

Below is the new Figure 2 on page 7 which merges the previously named Figures 3 and 4 (shown for easy reference below). Inset figures showing the suggested line scans are highlighted with a green box. The full-size Figure 2 can be found in the revised manuscript on page 7.

Previous Fig. 3

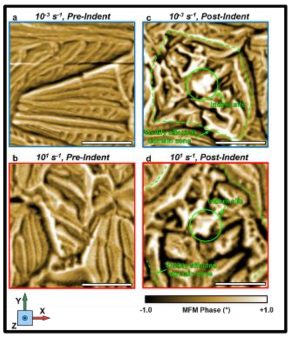

Previous Fig. 4

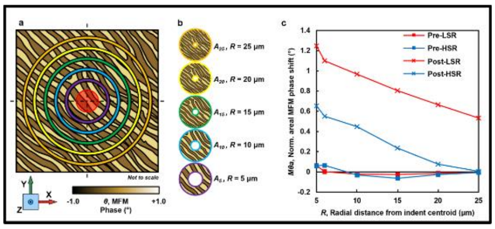

New Fig. 2

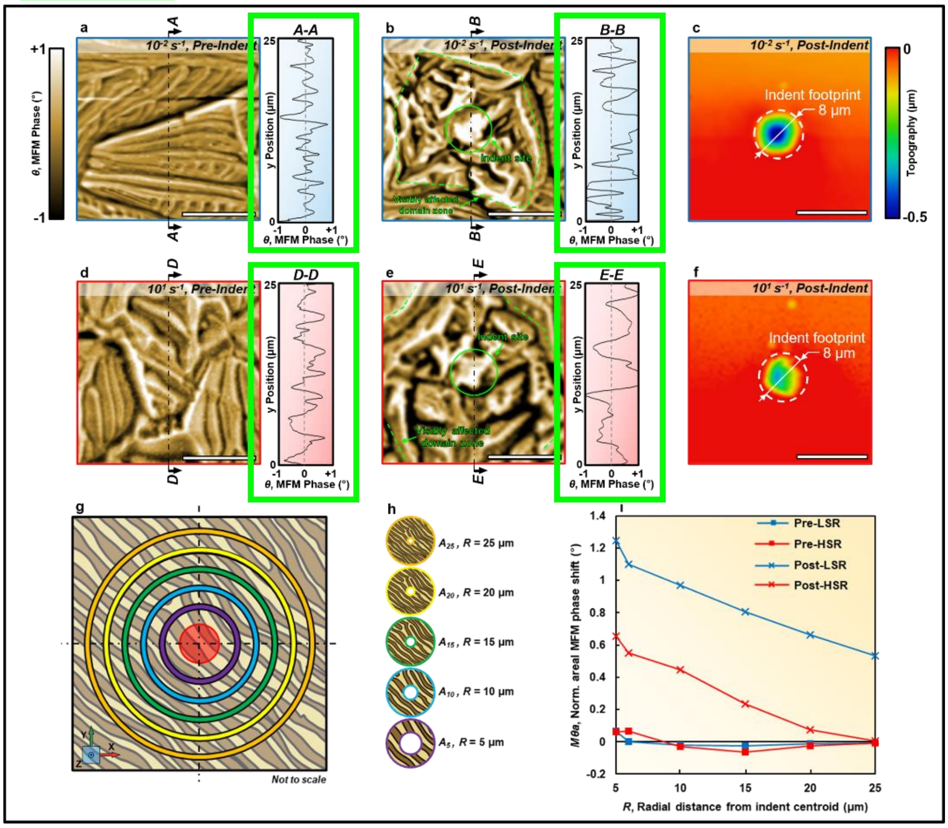

Figure 2 Magnetic domain disturbances caused by nano-indenter induced deformation within single FeSi grains <111>. a, d MFM images of the sample free-surface (||XY) taken prior to indenting at low (10^{-2} s^{-1}) and high (10^{1} s^{-1}) strain rates respectively, revealing the topographically independent base state of the magnetic domain structure. b, e MFM images taken post-indentation at low (10^{-2} s^{-1}) and high (10^{1} s^{-1}) strain rates respectively, in the same location as a, d, respectively. Line scans of the MFM Phase (θ) are shown adjacent to the maps a,b,d,e. Annotated areas in b, e indicate the indentation footprint (solid green circle) and the zone which visibly shows magnetic domain disruption surrounding the indent site (dashed green contour). c,f AFM topography maps of the surfaces shown in b,e, respectively, post-indentation. g,h Schematic representation of the annular regions extracted from the MFM images taken before and after indentation, used for calculating the mean areal MFM phase shift in i. The coloured rings in g correspond with the extracted areas in h of the same colours. The central red circle in g represents the indentation footprint, which is extracted from the calculation of the annular regions in h. i Plots of the mean areal MFM phase shift ($M\theta_A$) of the sample free-surface (||XY) before (square markers) and after (cross markers) indenting at low (10^{-2} s^{-1}, red lines) and high (10^{1} s^{-1}, blue lines) strain rates, revealing disparity in the magnetically disturbed areas – determined by the radial distance for the phase shift to reach the baseline (0°). MFM colour bar applies to subfigures a,b,d,e,g,h, indicating the centred MFM phase. Topography colour bar applies to sub figures c,f, indicating the surface height as measured from the mean surface plane. The coordinate system shown in g is the same for all subfigures. Scale bars in all subfigures, 10 µm.

Comment R3-8:

together with MFM images, topography images of the surface should be added. In particular because the topography may often induce electrostatic artifacts in MFM phase images and thus the possible morphology contribution to the MFM map should be discussed.

Response to comment R3-8:

Thank you for the suggestion. Topography images of the indented surface have been included in the newly consolidated Figure 2 on page 7. The authors have also taken the opportunity to discuss the potential morphology contribution to the data, as suggested by the reviewer. This text can be found in the revised manuscript on page 8 (copied below in amendments for comment R3-8, for reference).

The morphology contribution to the MFM map is controlled by using an interleave scan, where the topographic data was collected during the forward scan, enabling a constant lift height to be maintained between the tip and the surface during the lifted scan where MFM data was collected (new schematics of the MFM scan procedure have been included in the supplementary materials: S3 and is animated in Video 1). The MFM phase data at the lift height can be considered valid for relatively flat samples, however, when larger features are encountered (i.e., an indent) with non-planar surfaces, the tip encounters interactions from multiple directions which cannot be accounted for with the lift height alone – inducing artefacts in the MFM phase images. To account for this when quantitively measuring the radial extent of the magnetic domain disorder, we did not consider the MFM phase values within a diameter of 8 μm from the indentation centre (i.e., the indentation footprint which is commented on in **Comment R3-10**) so that the plots would refer to the affected area surrounding the indent.

Amendments in the revised manuscript on page 8 – Response to comments R3-8:

The surface morphology contribution to the MFM map was controlled by using an interleave scan, where the topographic data was collected during the forward scan for both LSR and HSR surfaces (Figure 2c, f respectively), enabling a constant lift height (100 nm) to be maintained between the tip and the surface during the lifted MFM scan (an animation visualising the forward and interleave scans is provided in the supplementary materials, S3). The MFM phase data at the lift height can be considered valid for relatively flat samples, however, when larger features are encountered (i.e., an indent which could reach micrometre level depths) with non-planar surfaces, the tip encounters interactions from multiple directions which cannot be accounted for with the lift height alone – inducing artefacts in the MFM phase images. To account for this, when quantitively measuring the radial extent of the magnetic domain disorder, we did not consider the MFM phase values within the indent footprint – the circular area with a diameter of 8 μm from the indentation centre. Therefore, we evaluated the changes in magnetic texture in the regions surrounding the indent footprint.

Amendments in the revised manuscript on page 7 – Response to comments R3-8:

Below is the new Figure 2 on page 7. Topography images of the indented surface are provided in subfigures c and f (these are highlighted below with a green box for reference, the full-size version of Figure 2 can be found in the revised manuscript on page 7). The so-called ‘indent footprint’ is annotated on these subfigures.

New Fig. 2

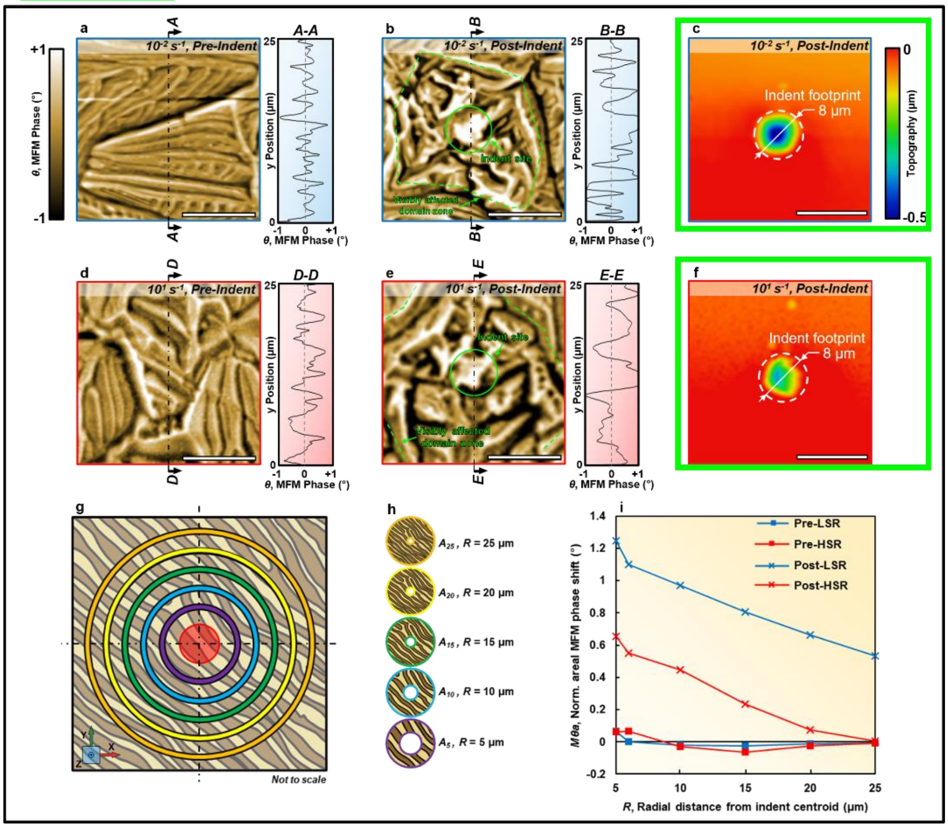

Figure 2 Magnetic domain disturbances caused by nano-indenter induced deformation within single FeSi grains <111>. a, d MFM images of the sample free-surface (||XY) taken prior to indenting at low (10^{-2} s^{-1}) and high (10^{1} s^{-1}) strain rates respectively, revealing the topographically independent base state of the magnetic domain structure. b, e MFM images taken post-indentation at low (10^{-2} s^{-1}) and high (10^{1} s^{-1}) strain rates respectively, in the same location as a, d, respectively. Line scans of the MFM Phase (θ) are shown adjacent to the maps a,b,d,e. Annotated areas in b, e indicate the indentation footprint (solid green circle) and the zone which visibly shows magnetic domain disruption surrounding the indent site (dashed green contour). c,f AFM topography maps of the surfaces shown in b,e, respectively, post-indentation. g,h Schematic representation of the annular regions extracted from the MFM images taken before and after indentation, used for calculating the mean areal MFM phase shift in i. The coloured rings in g correspond with the extracted areas in h of the same colours. The central red circle in g represents the indentation footprint, which is extracted from the calculation of the annular regions in h. i Plots of the mean areal MFM phase shift ($M\theta_A$) of the sample free-surface (||XY) before (square markers) and after (cross markers) indenting at low (10^{-2} s^{-1}, red lines) and high (10^{1} s^{-1}, blue lines) strain rates, revealing disparity in the magnetically disturbed areas – determined by the radial distance for the phase shift to reach the baseline (0°). MFM colour bar applies to subfigures a,b,d,e,g,h, indicating the centred MFM phase. Topography colour bar applies to sub figures c,f, indicating the surface height as measured from the mean surface plane. The coordinate system shown in g is the same for all subfigures. Scale bars in all subfigures, 10 µm.

Amendments in the revised manuscript in the supplementary materials S1 – Response to comments R3-8:

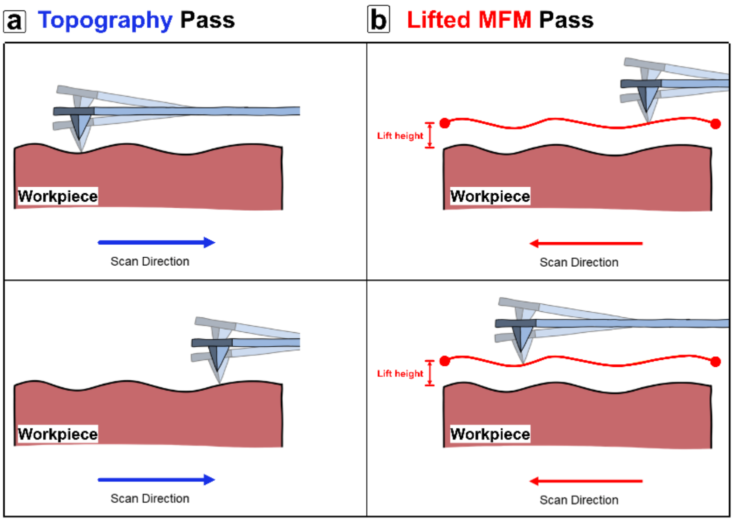

Figure 9 MFM mapping procedure. Frames from the supplementary animation of the MFM mapping procedure. Column a depicts the forward scan where the surface topography is mapped. Column b depicts the lifted scan where the cantilever and tip is raised to a set height above the surface measured in the forward scan, a. Note: Video 1 shows animated MFM scanning procedure.

Comment R3-9:

The authors report the roughness measured as to be 0.447 ± 0.0725 nm. It is not clear the importance of this parameter in the work. Also, if the information is maintained, please rationalize the significant digits of the mean and the uncertainty.

Response to comment R3-9:

Thank you for the comment. The authors agree that reporting the surface roughness to this precision is not practical, nor are the number of significant digits rational. The intention was to provide a value to the surface finish post polishing to demonstrate that the surface is free of significant topographic features which may introduce artefacts to the MFM phase measurements, however, this is already communicated when we state the final colloidal silica polishing step. Therefore, we have omitted the surface roughness value from the text.

Amendments in the revised manuscript on page 15 – Response to comments R3-9:

To understand the influence of indentation strain rate upon the magnetic domain texture, there is a need to start from a surface with minimal defects (e.g., scratches, surface roughness, and contamination). As such, prior to performing the indentations, a flat sample of a commercially available NOES (Arnon 7) was prepared by grinding and polishing with 0.04 µm colloidal silica ~~which resulted in high surface finish (Ra = 0.447 ± 0.07 nm)~~.

Comment R3-10:

the method for the analysis of the radial extent of the magnetic is interesting. However, some clarifications are needed, e.g., "The indent footprint itself was also removed from the annular subsets to avoid the inclusion of MFM…" what does it mean?

Response to comment R3-10:

Thank you for the question. The authors address this in the response to Comment R3-8. The removal of a circular area corresponding the physically deformed indentation area (the indent footprint) was used to mitigate the effects of the complex interactions between the tip and the non-uniform sample surface on the measurement of the radial extent of the magnetic disruption. The authors have clarified this in the revised manuscript on page 8 within the text that was also referenced in response to Comment R3-8.

Amendments in the revised manuscript on page 8 – Response to comments R3-10:

The surface morphology contribution to the MFM map was controlled by using an interleave scan, where the topographic data was collected during the forward scan for both LSR and HSR surfaces (Figure 2c, f respectively), enabling a constant lift height (100 nm) to be maintained between the tip and the surface during the lifted MFM scan (an animation visualising the forward and interleave scans is provided in the supplementary materials, S3 and Video 1). The MFM phase data at the lift height can be considered valid for relatively flat samples, however, when larger features are encountered (i.e., an indent which could reach micrometre level depths) with non-planar surfaces, the tip encounters interactions from multiple directions which cannot be accounted for with the lift height alone – inducing artefacts in the MFM phase images. To account for this, when quantitively measuring the radial extent of the magnetic domain disorder, we did not consider the MFM phase values within the indent footprint (represented by the red circular area in Figure 2g)– the circular area with a diameter of 8 µm from the indentation centre (shown in Figure 2c, f). Therefore, we evaluated the changes in magnetic texture in the regions surrounding the indent footprint.

Comment R3-11:

the method requires the integration over an circular region. Is the anisotropy relevant for this method? I mean, a large scale anisotropy due to the different grain orientation…

Response to comment R3-11:

Thank you for the comment. The MFM scans were completed within a single grains which were oriented with their <111> direction normal to the scanned surface. The radial measurements also were confined to a single grain – owing to the coarse-grained nature of the material. Therefore, the contribution of crystallographic anisotropy arising from bulk texture was minimised. We have provided a statistical plot of grain sizes for the material in the response to **Comment 2-7**.

Comment R3-12:

it is not clear which phase shift the authors use: is it the absolute shift or is the phase after subtraction of the mean (as it seems from Fig. 3)?

Response to comment R3-12:

Thank you for the question. The authors used the phase shift after subtraction of the mean. We have amended the text in the revised manuscript on page 6 to clarify this.

Amendments in the revised manuscript on page 6 – Response to comments R3-12:

The MFM maps plot the centred phase shift (θ) in the probe oscillation measured during the lifted scan. The centred phase shift (after subtraction of the mean) is used instead of absolute phase shift to account for any differences in the individual cantilever harmonics or tip magnetisation which may arise due to degradation of the consumable MFM tips.

Comment R3-13:

in supplementary materials, please note that MFM images are referred to as AFM.

Response to comment R3-13:

Thank you. The supplementary materials have been updated extensively and MFM maps are referred to as MFM instead of AFM. This is shown in the supplementary material (S1). The change is underlined in green below.

Amendments in the revised manuscript to the supplementary material, S1 – Response to comments R3-13:

Full sized figure can be found in the supplementary materials, S1, of the revised manuscript.

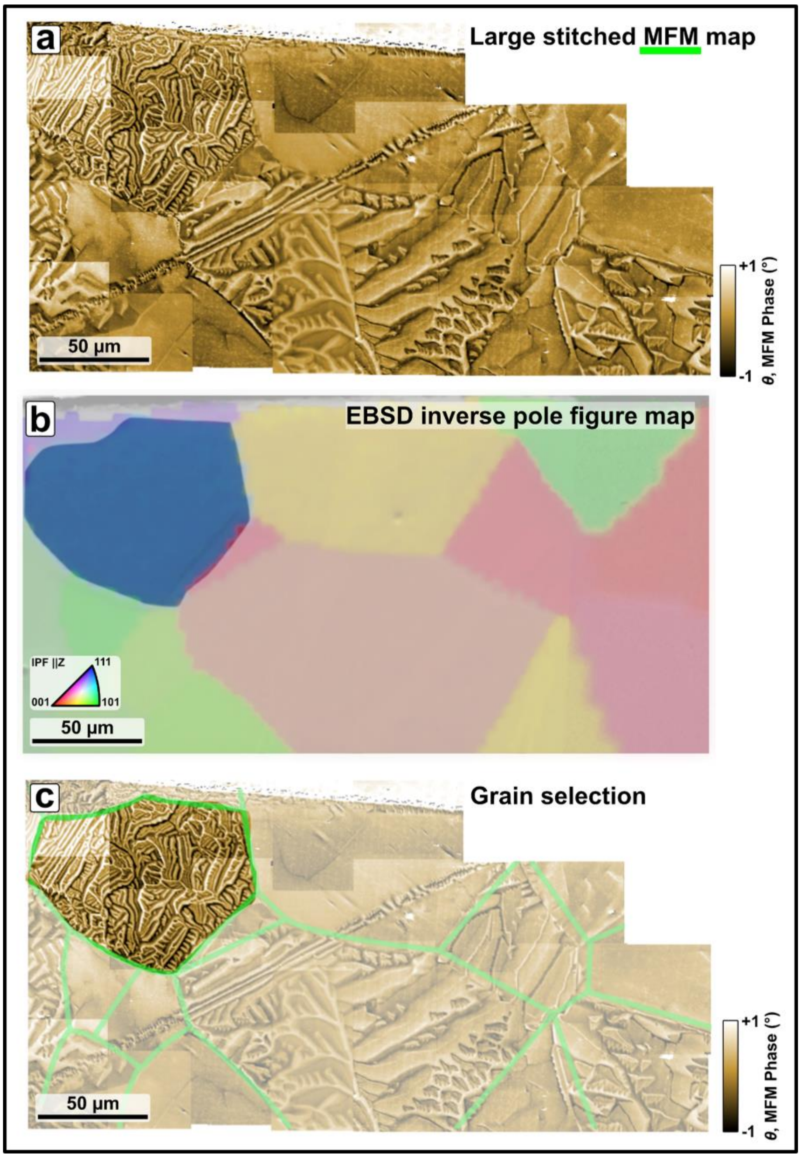

Figure 7 Nano-indentation grain selection procedure. a Large, stitched MFM map showing the MFM Phase, θ on the sample free-surface ||XY. b EBSD inverse pole figure (IPF) map of the same region mapped in a. The selected grain is highlighted and the colour corresponds to the stereographic triangle in the inset key. c Large, stitched MFM map as shown in a, with the grain boundaries measured in b superimposed to highlight the chosen grain. Colour scale for subfigures a, c, are located to the right of the respective maps. Scalebars for subfigures a-c, are located at the lower left of each map.

Response to Reviewer #4 (Amendments are marked in magenta):

Specific Comments:

Comment R4-1:

The re-arrangement of magnetic domains in a small area under stress/strain application has long been demonstrated (for example, Zhang et al., Nuclear Instruments and Methods in Physics Research B, 2007, 257, 379-382). This study, however, failed to further correlate plastic strain and domain re-arrangement. For example, high GROD values for the HSR sample are observed near the surface as compared to those for the LSR one, whereas higher MFM phase shifts are found for the latter.

Response to comment R4-1:

Thank you for taking the time to review our manuscript. The authors agree that the application of stress/strain has been demonstrated previously such as that referenced above in **Comment R4-1**, which investigates ion-induced strain application nano-crystalline Ni thin films and the resultant effect on magnetic domain rearrangement.

However, in our initial manuscript, we isolated the application of the load (through nano-indentation with a spherical tip) to within the boundaries of single grains of a known orientation (sample normal parallel with <111> oriented Fe-BCC crystals). To expand upon the pure application of strain, we also deformed the material at both low and high strain-rates to investigate the effects of dynamic loading upon the magnetic domain texture.

To further correlate plastic strain with domain rearrangement, we have added further experiments by compressing micro-pillars (at low and high strain rates) within a single grain of a known orientation (sample normal parallel with <100> oriented Fe-BCC crystals). This has enabled us to correlate the direction of loading, with the activation of preferential slip-systems during plastic deformation. The results from these additional experiments (Figure 4 in the revised manuscript) allows us to abstract the discussion and apply it to the MFM results to help explain the MFM phase shifts. The additional discussion relating to the correlation between the results obtained in the cross-section on page 11 of the revised manuscript is extracted and provided below. We have also made reference to the study given as an example in **comment R4-1** in the introduction on page 3 to include prior work in this field.

Amendments in the revised manuscript on page 11 – Response to comments R4-1:

Tying information on the magnetic domains in the cross-sectional (||XZ) observations presented in Figure 3 with those at the free-surface (||XY), shown in Figure 2, we can conclude that the strain-rate of applied deformation has a significant, time-dependent effect on the ability of the material to accommodate stresses. This has a marked effect on the magnetic textures at the level of a single grain. Applications of LSR loads were found to induce deformation which primarily altered the magnetic domain texture at the free-surface (||XY) – Figure 2i (see *Post-LSR* line). This may be attributed to the orientation of the activated primary slip system intersecting with the surface, allowing the domain pinning effects to be observed on the external surface. However, for HSR load application, occurring with an increase in indentation energy, non-preferentially aligned slip systems can be activated. In this case, the deformation boundaries which magnetic structures were observed to align with are situated deeper below the free-surface (||XY) (shown in the cross-section – Figure 3h). This had the effect of an apparent reduction in $M\theta_A$ on the free-surface (||XY), requiring cross-sectional observation to determine the arrangement of the magnetic structures – Figure 2i (see *Post-HSR* line).

Amendments in the revised manuscript on page 3 – Response to comments R4-1:

Prior work has also demonstrated the influence of plastic strain on the magnetic domain texture of nanocrystalline Ni thin films through Kerr microscopy and magnetic force microscopy [16], yet the strain-rate sensitivity of the magnetic response within a single crystal orientation should also be investigated.

Comment R4-2:

Moreover, the high GROD values mentioned above should result in a higher rotation of the magnetic moments. The projection of the saturated magnetization (Bs) on the sample surface and resulted change of magnetic deflection angle (beta L) should vary from one position to another on surface. However, the DPC map does not correspond to the

argument. Accordingly, this manuscript shows preliminary attempts to analyze local changes of magnetic domain structure on strain, but more efforts are needed to quantify the results.

<u>Response to comment R4-2:</u>

Thank you for your comments. To further the analysis on the changes of the magnetic domain structure due to the application of strain, we have completed additional experiments which have enabled us to better explain the results we observed in the initial nano-indentation experiments. We generated multiple micro-pillars within a single grain and compressed these similarly at low and high strain rates. Besides micro-pillars allowing for deformation which is not constrained by the bulk material (as in spherical nano-indentations), and applying the load with a constant projected area, we opted to load the crystal structure in a different direction (parallel to direction <100> as opposed to <111>) to investigate how orientation and subsequent slip system activation could influence the magnetic texture. These results are shown in the newly added Figure 4 in the manuscript. The accompanying discussion (pages 11-14) in the revised manuscript relating to the activation of slip systems relative to the direction of the applied load and the kinetic energy supplied to the system (at low and high strain rates) is also provided below.

Amendments in the revised manuscript on page 13 – Response to comments R4-2:

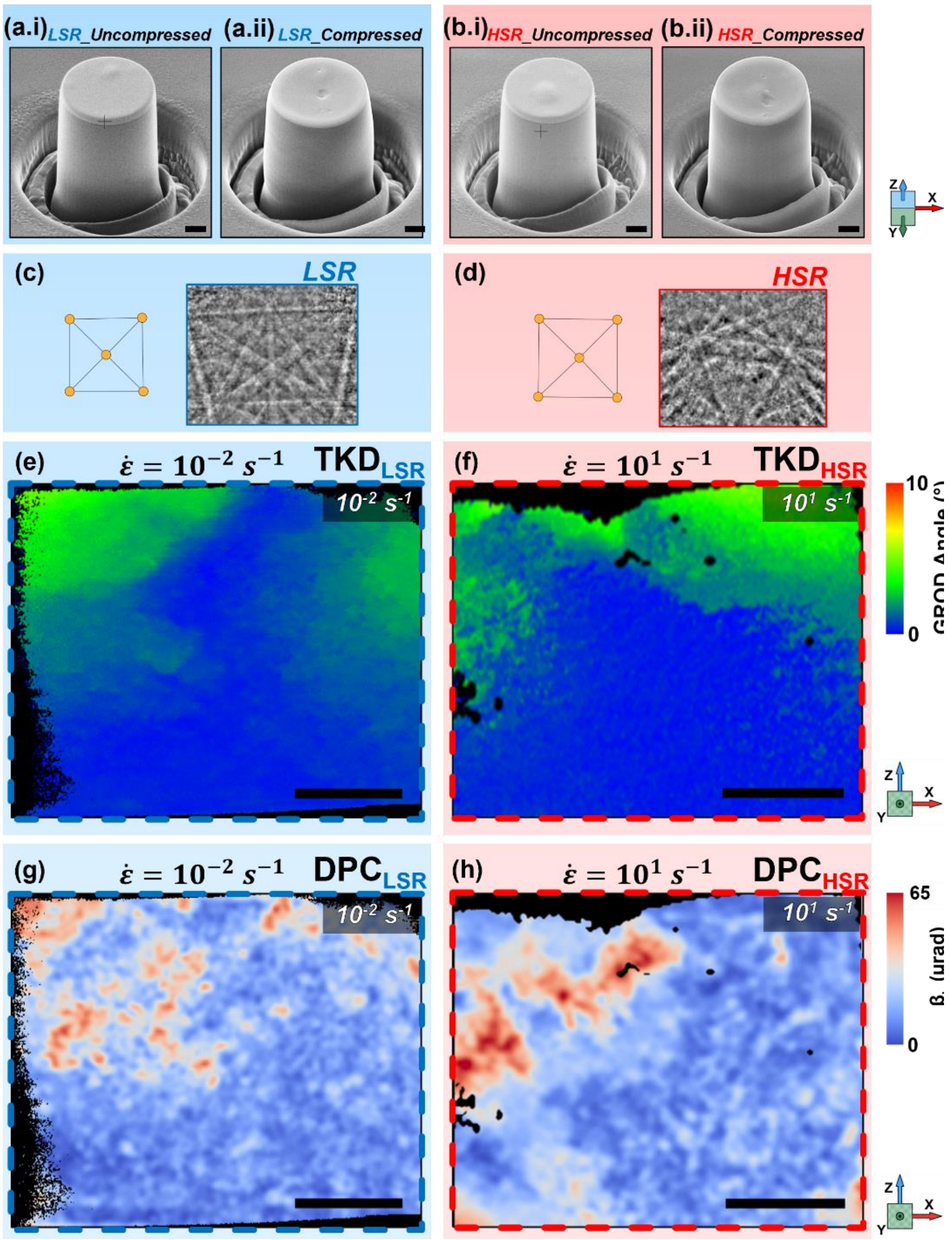

Figure 4 The effect of mechanically induced deformation on in-plane magnetic deflection within compressed pillars located in a single FeSi grain. a, b SEM images showing the pillar which was compressed at a low (0.01 s-1) and at a comparatively higher strain rate (10.00 s-1) strain rate before (a.i, b.i) and after (a.ii, b.ii) compression, respectively. c, d Extracted Kikuchi patterns and corresponding Fe-BCC unit cell representation from the cross-sectioned regions. e, f

TKD GROD angle maps revealing sub-granular deformation by means of crystallographic misorientation. The lamellae for e, f were extracted by cross-sectioning (in the ||XZ plane) the compressed pillars shown in a.ii, b.ii, respectively. Colour bar for both e, f indicates the GROD angle at the same scale. g, h STEM DPC images showing the in-plane (||XZ) magnetic deflection in the same compressed regions as in e, f, respectively. Colour bar for both g, h gives the magnitude of in-plane (||XZ) magnetic deflection angle, β_L, at the same scale. The coordinate systems for the subfigures are given by the triad on the right of each row. Scale bars in all subfigures a,b,e,f,g,h, 1 μm.

Amendments in the revised manuscript on page 11-12 – Response to comments R4-2:

To understand the effects that mechanically induced deformation at low and high strain-rates has on the magnetic texture, we performed scanning transmission electron microscopy – differential phase contrast (STEM-DPC) (Figure 3g, h) with a pixellated detector to map the magnitude of the in-plane magnetic deflection, β_L, beneath the indentations (see more details in the methods section).

In LSR indentation, plastic deformation is focussed on a lower number of primary slip systems, which require a lower energy threshold for activation compared to the secondary slip systems [28]. As the grain was loaded in the <111> direction, the {111} family of slip planes (the primary slip planes in BCC crystals) were easily activated. The more vertically oriented bands observed in the DPC map (Figure 3g) suggests a correlation between the direction of the deformation network and the arrangement of magnetic structures.

Conversely, in HSR indentation, the activation energy conditions for multiple, secondary slip systems may be activated during dynamic loading. The activation of these secondary slip systems causes diffusion of the plastic deformation, which leads to directionally shifted magnetic structures observed in the two-dimensional cross-section view (Figure 3h). As the dynamic HSR indentation with a greater input energy can activate the secondary slip systems deeper below the indent surface, horizontal contours in the TKD map (Figure 3f) have been observed. Despite the region immediately below the indentation surface having a greater magnitude of deformation than that which occurred deeper, the corresponding DPC map (Figure 3h) highlights that there is a stronger relationship between the direction of the activated slip systems and the magnetic alignment than with the deformation magnitude. This activation of non-primary slip systems in the HSR sample, coupled with the simultaneous occurrence of both shearing and compression during fast deformation events gives rise to a greater number of boundaries at which the magnetic structures can accumulate.

Tying information on the magnetic domains in the cross-sectional (||XZ) observations presented in Figure 3 with those at the free-surface (||XY), shown in Figure 2, we can conclude that the strain-rate of applied deformation has a significant, time-dependent effect on the ability of the material to accommodate stresses. This has a marked effect on the magnetic textures at the level of a single grain. Applications of LSR loads were found to induce deformation which primarily altered the magnetic domain texture at the free-surface (||XY) – Figure 2i (see *Post-LSR* line). This may be attributed to the orientation of the activated primary slip system intersecting with the surface, allowing the domain pinning effects to be observed on the external surface. However, for HSR load application, occurring with an increase in indentation energy, non-preferentially aligned slip systems can be activated. In this case, the deformation boundaries which magnetic structures were observed to align with are situated deeper below the free-surface (||XY) (shown in the cross-section – Figure 3h). This had the effect of an apparent reduction in $M\theta_A$ on the free-surface (||XY), requiring cross-sectional observation to determine the arrangement of the magnetic structures – Figure 2i (see *Post-HSR* line).

Amendments in the revised manuscript on page 12-14 – Response to comments R4-2:

To provide more evidence for the observations discussed around the changes of magnetic domains within plastically deformed regions, we have performed further tests in different loading conditions. To simplify the loading scenario, and to avoid a complex stress distribution akin to that which occurred in the conventional spherical indentation (as above), we compressed a set of micro-pillars (Figure 4a.i, b.i – pre-compression, Figure 4a.ii, b.ii – post-compression at LSR and HSR respectively) which were located within a single grain oriented with the <100> direction of the crystal parallel to the defined X, Y, and Z axes. Compressing pillars ensures that the loaded area of the grain, hence the magnetic texture, is isolated from the neighbouring material. The pillars were deformed plastically to the same strain ($\varepsilon = 0.078$) at opposing strain rates. Like the spherical indents, we compressed the pillars plastically at both LSR (10^{-2}) and HSR (10^{1}) to isolate and provide more evidence to how the different deformation mechanisms influence the distribution of magnetic textures. To further support the discussions referring to slip system activation when referring

to the changes in magnetic textures, we performed LSR and HSR tests on the pillars at a different crystal orientation (<100>) from that used in the previously discussed spherical indentations (<111>). As such, we would not expect the deformation observed from TKD nor the magnetic alignment to occur in the same directions.

For both LSR (Figure 4e) and HSR (Figure 4f) pillar compression, the deformation from the TKD maps predominantly aligns with the primary slip planes in the <111> direction. The corresponding DPC map for LSR pillar compression (Figure 4g) shows a weak banded magnetic structure following this alignment indicating that, like in the quasi-static indentations (LSR), the plastic deformation can accumulate along the easily activated primary slip planes. However, as the pillar compression energy increases at HSR, secondary slip systems can be activated, inducing alignment in different directions, resulting in a more-complex deformation network (Figure 4f). This activation of secondary slip systems at HSR (Figure 4h) presents more boundary options for the magnetic structures to align with.

The authors would like to thank the reviewers for their valuable feedback to improve the quality of our submission.

Reviewer Comments:

Reviewer #3 (Remarks to the Author):

I thank the Authors for how they addressed the comments. I think the manuscript can be published as it is.

Reviewer #5 (Remarks to the Author):

The micro-pillar work was greatly appreciated. It greatly reduce the stress complexity as compared to spherical indentation tests therefore the effects of crystal orientation and strain-rate can be discussed more clearly.

Just a few more questions and comments on how magnetic deflection relates to micro-deformation:
1. In Fig. 4(c) and (d), the Kikuchi patterns of the lamellar show different orientation. What is the explanation for this if the lamellar were all FIBBed from the center of the pillarr

2. The author used the concept that HSR tests provide higher energy hence able to activate more slip systems; I found this somehow misleading and need to be corrected. Plasticity in BCC metals are thermally-activated and strain-rate dependent, e.g. BCC metals exhibit higher plasticity at elevated temperatures and lower strain-rate. The thermally-activated dislocations are carrier of plasticity, if provided with sufficient time. This can be seen in GROD maps from HSR tests in Fig. 3(f) and Fig. 4(f), where higher amount of deformations (higher GROD value) accumulated at the surface since mobile dislocations lack sufficient time to move outwards, relieving the accumulated stresses. Just to be clear, the Peierls stress for the dislocation motion on the activated slip system are the same for LSR and HSR tests, therefore HSR tests will NOT activate additional slip systems since crystal orientation and temperature of the two test scenario are the same.

3. The usage of TKD techniques can also be quite misleading, as the stress-state are far from the as-compressed state due to the thinning. Can the author provide non-TKD configuration (clean pillar surface using FIB) for GROD calculation? There are some very nice work done by Kalacska et al. on characterizing micro-deformations of Cu micro-pillars (MSEA, 770(7), 2020). Her work was also done in conventional EBSD mode so should be a nice reference for calculation the deformation underneath the Fe-3Si micro-pillar.

Response to Reviewer #3:

General comments:

I thank the Authors for how they addressed the comments. I think the manuscript can be published as it is.

Response to general comments:

Thank you for taking the time to review this iteration of our manuscript.

Specific comments:

N/A

Response to Reviewer #5 (Amendments are marked in cyan):

General comments:

The micro-pillar work was greatly appreciated. It greatly reduce the stress complexity as compared to spherical indentation tests therefore the effects of crystal orientation and strain-rate can be discussed more clearly.

Just a few more questions and comments on how magnetic deflection relates to micro-deformation:

Response to general comments:

Thank you for taking the time to review our manuscript and for providing constructive comments.

Specific Comments:

Comment R5-1:

In Fig. 4(c) and (d), the Kikuchi patterns of the lamellar show different orientation. What is the explanation for this if the lamellar were all FIBBed from the center of the pillar?

Response to comments R5-1:

Thank you for the constructive question. The Kikuchi patterns presented in Figure 4 show the same overall orientation (with a small amount of rotation present likely from the lift-out and attachment to the Omniprobe grid). The difference between the images is that the pattern centre on Fig. 4(d) is higher than in Fig. 4(c). In Figure R5-1.1 below, a yellow triangle has been added to each subfigure to bring attention to the same regions of the pattern.

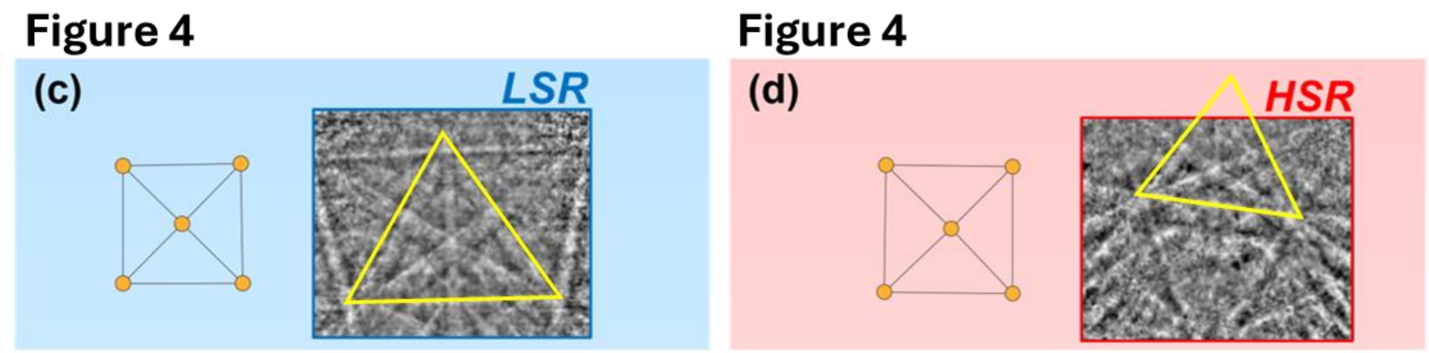

Figure R5-1.1: Extracted subfigures c and d from figure 4 in the manuscript. The selected Kikuchi patterns are shown. A yellow triangle has been appended on each to show the comparative points.

The pattern centre is higher in Fig. 4(d) because the elevation of the EBSD camera was at a value of -3.0 mm compared to -2.0 mm for the Kikuchi pattern in Fig. 4(c). There was some suspected shadowing from the TKD holder at the top of the Kikuchi pattern when completing the mapping corresponding to the pattern in Fig. 4(d). Therefore, decreasing the elevation 'raises' the Kikuchi pattern image as shown below in Figure R5-1.2. Indexing was still possible in this position as sufficient bands were present.

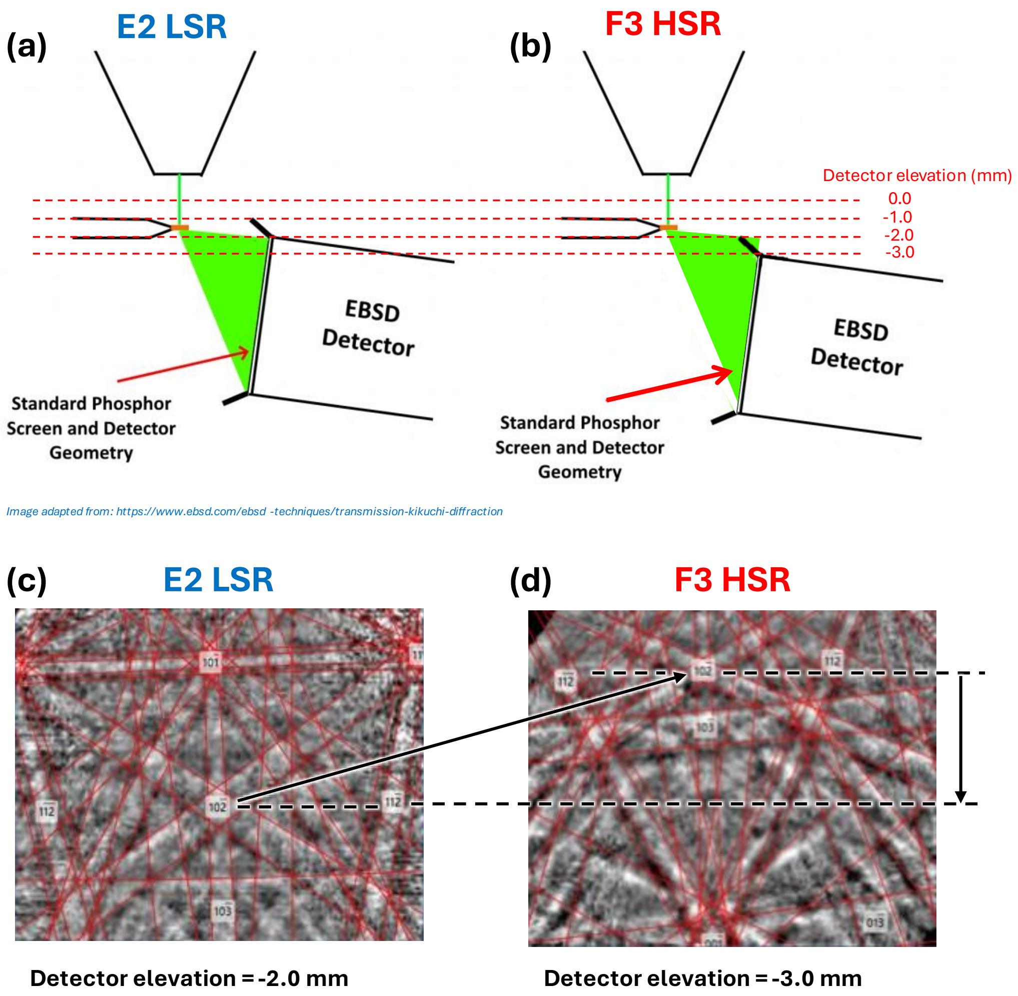

Figure R5-1.2: Simplified schematic of the EBSD detector geometry relative to the detector elevation for the TKD scans at (a) LSR (corresponding to manuscript Fig. 4(c)) and (b) HSR (corresponding to manuscript Fig. 4(d)). Further examples of extracted Kikuchi patterns taken from the same TKD scans confirming the same orientation is shown in (c) and (d) of this figure with arrows indicating the change in map position due to the detector elevation.

Comment R5-2:

The author used the concept that HSR tests provide higher energy hence able to activate more slip systems; I found this somehow misleading and need to be corrected. Plasticity in BCC metals are thermally-activated and strain-rate dependent, e.g. BCC metals exhibit higher plasticity at elevated temperatures and lower strain-rate. The thermally-activated dislocations are carrier of plasticity, if provided with sufficient time. This can be seen in GROD maps from HSR tests in Fig. 3(f) and Fig. 4(f), where higher amount of deformations (higher GROD value) accumulated at the surface since mobile dislocations lack sufficient time to move outwards, relieving the accumulated stresses. Just to be clear, the Peierls stress for the dislocation motion on the activated slip system are the same for LSR and HSR tests, therefore HSR tests will NOT activate additional slip systems since crystal orientation and temperature of the two test scenario are the same.

Response to comments R5-2:

Thank you for the constructive critique and highlighting the need for clarification regarding the activation of slip systems in high strain-rate (HSR) tests. The authors appreciate your insights into the behaviour of BCC metals under different conditions which have enabled us to update our discussion in the manuscript accordingly.

The authors agree with the comments regarding plasticity in BCC metals being thermally activated and strain-rate dependent, and that the Peierls stress for dislocation motion remains constant for both LSR and HSR conditions for the same crystal orientation and temperature.

In the previous revision of the manuscript, the author's intention was to convey that HSR tests, due to their rapid loading nature, could lead to different deformation mechanisms compared to LSR tests. In mentioning 'additional slip systems' previously, the intention was to propose how the dislocation avalanche was able to propagate along a greater number of crystallographic directions present within the same, primary, slip system of the Fe-BCC crystal that have a lower

resolved shear stress, as opposed to the activation of additional, secondary, slip systems. The authors acknowledge that the explanation was not clear and somewhat misleading, and we have corrected the discussion accordingly.

Specifically, we have clarified the impact of strain-rate: where at higher strain-rates, the rapid accumulation of stress can result in the accumulation of dislocations near the surface, as there is insufficient time for mobile dislocations to move outwards. We have also made clarifications to the deformation mechanisms discussed: while the Peierls stress remains unchanged, the rapid deformation associated with HSR conditions can influence the organisation and density of dislocations, leading to different stress states and dislocation interactions compared to LSR. This can contribute to the variations in magnetic domain structure which was observed in the experiments.

Thank you again for your valuable feedback which has improved the accuracy and clarity of our manuscript. We have updated the abstract in the manuscript on page 1 to clarify the changes. We have also updated the discussion in manuscript on pages 11-14 to clarify that HSR tests do not activate additional slip systems, but rather lead to different deformation dynamics due to the constrained time for dislocation movement. We have also modified the conclusions to reflect these changes on page 21.

Amendments in the abstract of the revised manuscript on page 1 – Response to Reviewer #5 general comment R5-2:

Our analysis, employing magnetic force microscopy, transmission Kikuchi diffraction, and scanning transmission electron microscopy with a pixelated detector, reveals that magnetic texture disturbances rely on the time-dependent dislocation dynamics of the Fe-BCC material.

Amendments in the discussion of the revised manuscript on pages 11-14 – Response to Reviewer #5 general comment R5-2:

… In LSR indentation, plastic deformation occurs primarily along the primary slip systems, which are easily activated under quasi-static conditions[30]. As the grain was loaded in the <111> direction, the {110} family of slip planes (the primary slip planes in BCC crystals) were easily activated. …

… Conversely, in HSR indentation, the deformation occurs rapidly, allowing only a limited time for dislocations to move and rearrange. This results in a unique arrangement of dislocations compared to LSR indentation. While the Peierls stress, which determines the resistance to dislocation motion, remains constant (i.e., is independent of strain-rate), the rapid nature of HSR indentation increases dislocation interactions and density – especially near to the indentation surface, due to constrained mobility. This results in localised stress concentrations and limits the volume for plastic deformation to diffuse into, leading to directionally altered magnetic structures, as seen in the two-dimensional cross-section view (Figure 3h). Despite the region immediately below the indentation surface having a greater magnitude of deformation than that which occurred deeper, the corresponding DPC map (Figure 3h) highlights that there is a stronger relationship between the dislocation network's direction and the magnetic alignment than with the deformation magnitude. The differences in deformation behaviour between HSR and LSR highlight the time-dependent nature of dislocation motion and interactions during rapid loading, significantly affecting both the mechanical and magnetic properties of the material. …

… However, for HSR load application, the rapid deformation results in a different dislocation arrangement rather than the activation of additional slip systems as the temperature remains constant for both LSR and HSR indentation. In this case, the deformation boundaries, with which magnetic structures were observed to align, are situated deeper below the free-surface (||XY) (shown in the cross-section - Figure 3h). …

… The corresponding DPC map for LSR pillar compression (Figure 4g) shows a weakly banded magnetic structure, indicating that plastic deformation accumulates and organises along the easily activated primary slip planes, as expected under quasi-static conditions. However, the rapid deformation in HSR pillar compression leads to a denser and more complex dislocation network (rather than the activation of secondary slip systems) near the surface. The limited time for dislocations to move and reorganise under HSR conditions results in disordered interactions along dislocations, which in turn create complicated stress fields. This increased dislocation density and complexity provide additional boundaries and pathways for magnetic alignment, as seen in (Figure 4h). …

Amendments in the conclusion of the revised manuscript on page 21 – Response to Reviewer #5 general comment R5-2:

… We demonstrate that by mechanically inducing deformation at low (10^{-2} s^{-1}) and high (10^{1} s^{-1}) strain-rates within a single grain, the magnetic domain textures can be significantly altered. These changes are primarily due to the time-dependent dislocation dynamics and the resulting stress fields, which introduces pinning sites that can hinder magnetic domain wall motion during subsequent magnetisation. …

… This could be due to the activation of primary slip systems in a quasi-static conditions, which allows plastic deformation to accumulate along the activated planes in an organised manner as there is sufficient time for the dislocations to be mobile. In contrast, the deformation at higher strain-rates is more rapid, which leads to a more complex dislocation network, resulting in increased dislocation density and interactions near the free surface (||XY). This creates additional boundaries and pathways for magnetic structures, affecting the alignment and behaviour of magnetic domains therefore, there can be less visible preferential alignment in the 2D cross-section view. …

Comment R5-3:

The usage of TKD techniques can also be quite misleading, as the stress-state are far from the as-compressed state due to the thinning. Can the author provide non-TKD configuration (clean pillar surface using FIB) for GROD calculation? There are some very nice work done by Kalacska et al. on characterizing micro-deformations of Cu micro-pillars (MSEA, 770(7), 2020). Her work was also done in conventional EBSD mode so should be a nice reference for calculation the deformation underneath the Fe-3Si micro-pillar.

This is excellent work so I'd appreciate using the suitable dislocation-based mechanics to explain plasticity, and additional work for characterizing the micro-deformations using EBSD technique.

Response to comments R5-3:

Thank you for the very helpful comment and for highlighting this impressive work by Kalacska et al. to our attention. The authors agree that thinning the pillar cross-section in the compressed state may lead to dislocation relaxation - which can lead to an apparent reduction in dislocation density in the TKD state.

Due to this, the authors had opted to plot 'deformation' in terms of GROD angle as opposed to the GND. With this approach we intended to produce images which show the relative position and intensity/magnitude of the dislocation network (albeit at an apparent reduced magnitude due to the relaxation) so that the lower and higher strain rate compressions could be compared to each other only.

We would like to reiterate that, although following the referenced methodology by Kalacska et al. would definitely improve our work by enabling the actual dislocation distribution to be measured with greater accuracy, for the current manuscript - the complexity would put these tests out of scope. For EBSD geometry, the pillars must be situated at the edge of the sample. The grain selected for the micro-pillars is quite central to the sample. In order to replicate the micro-pillar manufacture, compression and then compare the results using EBSD geometry to the TKD method, a large-scale bulk lift-out would be required to position the pillars on a 90° edge which would increase the risk of damage/loss of the sample.

For future studies, however, we can certainly see the advantage in classifying the dislocation structures 3-dimensionally with the appropriate sample geometry. We have made an addition to the manuscript on page 18 to reflect these considerations and have referenced the mentioned study by Kalacska.

Amendments in the methods section of the revised manuscript on page 18 – Response to Reviewer #5 general comment R5-3:

To map the relative sub-granular deformation occurring beneath the indentations and compressed pillars, off-axis TKD was performed with an accelerating voltage of 30 kV, an electron beam current of 1.0 nA, and a pixel size of 0.02 µm. These parameters allowed us to obtain a high level of angular precision for mapping the grain-referenced orientation deviation (GROD) angle for each scan-point in the crystal lattice beneath the indentations and compressed pillars. It should also be noted that the act of thinning the deformed regions (indentations and compressed pillars) into lamellae may cause some dislocation relaxation – which could lead to an apparent reduction in the dislocation density when measured with TKD. A solution to this problem has been proposed and explored successfully by Kalácska et al. [32] where serial FIB slicing was used on deformed, square, copper micro-pillars to observe the dislocation network in 3D to obtain more reliable results on the geometrically necessary dislocation (GND) density. However, this would require the grain of interest to be positioned on the edge of the sample, and the method is destructive – limiting further analysis of the sample with STEM methods, placing this method outside the scope of the current study.
